# The most significant change for Colombian medical trainees going transformative learning on cultural safety: qualitative results from a randomised controlled trial

**DOI:** 10.1186/s12909-022-03711-1

**Published:** 2022-09-10

**Authors:** Juan Pimentel, Paola López, Anne Cockcroft, Neil Andersson

**Affiliations:** 1 CIET-PRAM, Department of Family Medicine, McGill University, 5858 Chemin de la Côte-des-Neiges 3rd Floor, Suite 300, Montreal, QC H3S 1Z1 Canada; 2 Facultad de Medicina, Universidad de La Sabana, Campus Universitario puente del común, CP 250001 Chía, Colombia; 3 Centro de Investigación de Enfermedades Tropicales (CIET), Universidad Autónoma de Guerrero, Calle Pino S/N Colonia El Roble, 39640 Acapulco, Guerrero Mexico

**Keywords:** Cultural safety, Medical education, Colombia, Transformative learning, Most significant change

## Abstract

**Background:**

Cultural safety training is not yet standard in Colombian medical education. If incorporated, it could address currently adversarial interactions between health professionals and the 40% of people who use traditional medicine practices. In 2019, a randomised controlled trial tested the impact of cultural safety training for medical students using participatory serious game design. The quantitative evaluation showed improved cultural safety intentions of Colombian medical trainees. We report here a qualitative evaluation of the most significant change perceived by trial participants.

**Methods:**

This qualitative descriptive study used the most significant change technique. We invited the trial participants engaged in clinical settings to describe stories of change in their supervised clinical practice that they attributed to the intervention. Using a deductive thematic analysis based on a modified theory of planned behaviour, two independent reviewers coded the stories and, by consensus, created themes and sub-themes.

**Results:**

From 27 stories of change, we identified seven themes and 15 subthemes: (a) Conscious knowledge: benefits of cultural safety training, consequences of culturally unsafe behaviour, cultural diversity and cultural practices; (b) Attitudes: respect and appreciation for cultural diversity, openness, and self-awareness; (c) Subjective norms: positive perception of cultural practices and less ethnocentrism; (d) Intention to Change; (e) Agency to accept cultural diversity and to prevent culturally unsafe actions; (f) Discussion; and (g) Action: better communication and relationship with patients and peers, improved outcomes for patients, physicians, and society, investigation about cultural health practices, and efforts to integrate modern medicine and cultural health practices.

**Conclusion:**

The narratives illustrated the transformative impact of cultural safety training on a results chain from conscious knowledge through to action. Our results encourage medical educators to report other cultural safety training experiences, ideally using patient-related outcomes or direct observation of medical trainees in clinical practice.

**Trial registration:**

Registered on ISRCTN registry on 18/07/2019. Registration number: ISRCTN14261595.

**Supplementary Information:**

The online version contains supplementary material available at 10.1186/s12909-022-03711-1.

## Background

The Colombian government and private institutions provide health services based on Western biomedical principles. Some 40% of the population, however, seek care in traditional health practices [1], defined as the practices based on the theories indigenous to different cultures, used to improve health [2]. This divide illustrates a gap between the expectations of the population, who use traditional medicine, and the skills of health professionals, who have no training to interact with traditional medicine users. The resulting intercultural tensions in clinical practice hinder full access to health services for those who use traditional and cultural health practices [3], exacerbating health disparities in an already inequitable setting -Colombia has worse health inequalities than the world average- [4].

There is ample evidence that respecting culture in medical education leads to benefits in patient satisfaction and adherence to treatment, mutual understanding between health professionals and patients from non-dominant cultures -broadly cultures other than those who identify as European origin-, improved knowledge, attitudes, and skills of trainees, and enhanced respect for and acceptance of traditional and cultural health practices [5–8]. This type of medical education, however, has not spread beyond high-income countries like Australia, New Zealand, Canada, and United States [8]. In culturally diverse Latin America, there is arguably an even greater need to train health professionals to provide care that is congruent with the cultures of the people they serve.

### Cultural safety training in Colombia

Cultural safety can be “a space that is spiritually, socially, emotionally and physically safe for people; where there is no assault, challenge or denial of their identity, of who they are, and what they need.”(p213) [9]. The foundation of cultural safety is that it depends on people, including patients, from non-dominant cultures engaging in equal partnerships in clinical practice, to protect their cultural identity and well-being [10]. Perceptions of patients and communities are pivotal in cultural safety, distinguishing it from cultural sensitivity and cultural competence which are usually criteria set from within the dominant culture [11, 12].

Over recent years, we pioneered a new approach to cultural safety training in Latin America [13, 14]. In preparing the curriculum, we reported final-year medical students’ positive perception of traditional and cultural health practices after participating in a five-month community-based cultural safety training intervention. In 2019, a randomised controlled trial (RCT) tested the training with Colombian medical students and medical interns at *La Sabana* University in Chia, just north of Bogotá in Colombia [15, 16]. The trial tested an intervention based on transformative learning [17], using a format that was less time- and resource-consuming than 5-month community-based learning.

Since it was unrealistic to assess patient-related outcomes [5] for current medical students, the quantitative assessment relied on education-related outcomes, such as self-reported intended behaviour and self-confidence in transcultural skills. We complemented our quantitative evaluation with a narrative exploration of the impact of cultural safety training on the clinical practice of the Colombian trainees. In this article, we present the self-reported most significant change in supervised clinical practice attributed to the participation in the trial by Colombian medical students and medical interns.

## Methods

### Study design

Our parallel-group, two-arm RCT evaluated whether medical student participation in a game jam on cultural safety is more effective than a standard lesson plus interactive workshop in changing self-reported intended patient-oriented behavior. Six months after the intervention and control activities, we conducted this narrative assessment of the impact of the trial. For this qualitative exploration, we only invited students who participated in the trial. The complete protocol (Additional file 1) [15] and quantitative results of the trial [16] are available.

We used the Most Significant Change technique (MSC) [18] for the narrative evaluation because the goal of cultural safety training is attitudinal and behavioural change of service providers, rather than merely knowledge acquisition [19]. MSC allows people to report changes meaningful to them after engaging in an intervention [18]. Our report follows the Standards for Reporting Qualitative Research (Additional file 2) [20].

### Setting and participants

We conducted the RCT at *La Sabana* University in Chia, Colombia, which is located 20 min north of Bogota, the capital of the country. *La Sabana* is a private university providing education to close to 9,000 undergraduate students, including 956 medical students and 256 medical interns [21]. Our anticipated sample size was 199 students in each trial arm (*n* = 398) [15]. We used stratified randomization to allocate the participants to the intervention and control groups. The inclusion criteria for the trial were giving informed consent and being a medical intern or medical student at any level of training. We did not include trainees who did not agree to participate in the study.

### Interventions

Transformative learning requires education that is participatory and interactive, through group problem-solving or communicative learning [22], all of which are present in *game jams*. Game jams are participatory events that allow attendees to create games in a time-restricted environment [23]. The intervention was a game jam to create a prototype of an educational game on cultural safety. The activity included: (a) preliminary lecture on cultural safety [24] and game design; (b) game building session where groups of participants created educational games about cultural safety; and (c) play-test session in which participants played and learned from each other’s games. The lecture was based on our co-designed curriculum [25] and provided key elements of cultural safety, including (a) the definition of cultural safety; (b) consequences of cultural tensions in health care; (c) self-awareness; (d) Colombian cultural health practices; and (e) respect for patients who use traditional and cultural health practices.

The control group received a PowerPoint-based standard lecture on cultural safety plus an interactive workshop over several hours in which groups of participants created infographics to communicate selected readings on cultural safety. The control group received the same key concepts of cultural safety based on our previously co-designed curriculum [24] as the intervention group. The duration of both the intervention and control group activities was the same (eight hours). A detailed description of these activities is available [15].

### Data collection

For the narrative assessment, we used a pre-defined format in Google forms and asked participants to write and upload their stories of change. We used three questions: *(a) what do you believe was the most significant change in your clinical practice as a result of your participation in the activity [game jam or standard lesson] 6 months ago? (b) can you please share a real-life story depicting this change? (c) why do you think this story is significant?* We submitted an email with the link to the online form and collected the narratives of change in both the intervention and control groups six months after the intervention, from January to May of 2020.

The instructions made clear that participants were free to write stories of negative changes or to say that they did not experience any change at all, and stressed that the stories would not have any influence in the avaluation of their performance in the faculty of medicine. The format was completely anonymous; it did not collect any personal information from the participants. In this qualitative assessment we were not interested in comparing the impact between intervention and control groups. Rather, we were interested in describing the perceptions of the students after receiving cultural safety training based on our co-designed curriculum [24]. From the 531 students who originally participated in the trial, we invited only medical students involved in supervised clinical practice and medical interns (third to seventh year of medical school) to participate in this part of the RCT (*n* = 412).

### Data processing and analysis

We used AtlasTi 8 to support our analysis of the stories. Two researchers (JP and PL) individually coded the transcripts of the anonymized stories following a deductive thematic analysis approach. They later compared their analysis and, by consensus, decided on themes and subthemes. In deductive analysis, a theory aligned with the researchers’ interest drives the data analysis [26]. Examples of theories used in deductive analysis include the transtheoretical model [27], and the knowledge, attitudes and practices model [28]. We used the intermediate outcomes of the CASCADA model [29] to identify themes of change in the stories. The model includes the elements of a modified theory of planned behaviour [30, 31]: Conscious knowledge, Attitudes, Subjective norms, Intention to Change, Agency, Discussion, and Action or behaviour change. The two researchers held ten two-hour meetings to carry out the qualitative analysis.

### Rigour

We adhered to the strategies for ensuring trustworthiness in qualitative research suggested by Patton [32] and Shenton [33]. We increased credibility by using validated research methods to collect (MSC) and standard deductive thematic analysis to process the data. We enhanced dependability by adhering to the Standards for Reporting Qualitative Research and by publishing the protocol of our study in advance [15], which will allow researchers to replicate the study in the future. We increased confirmability by disclosing the background of the researchers directly involved in the data analysis, as well as by recognizing the limitations of the study. JP, an MD with an MSc in Epidemiology and a Doctoral Candidate in Family Medicine, guided PL during the analysis phase. PL was a fourth-year medical student with no previous experience in qualitative research or cultural safety when this article was written. Before the analysis, JP provided PL a two-day training on qualitative research and thematic analysis.

### Ethics

The Institutional Review Board of the McGill’s Faculty of Medicine (approval number A05-B37-17B) and the Sub-committee for Research of the Faculty of Medicine at *La Sabana* University (approval number 445) provided ethical clearance for this study. All participants signed informed consent before proceeding with any research activity.

## Results

Some 531 students (366 females) completed the baseline survey and were randomised. A complete description of the sociodemographic characteristics of the RCT participants is available [16]. For the qualitative assessment, we received 27 stories of change from the 412 invited participants. A deductive thematic analysis identified themes aligned with the seven elements of the CASCADA model and 15 subthemes (Table 1 and Additional file 3).

**Table 1:** Themes and subthemes of the deductive thematic analysis

Theme	Subtheme
Conscious knowledge	⦁ Benefits of cultural safety training and consequences of cultural risk ⦁ Acknowledging cultural diversity and traditional medicine use among family, setting, and students ⦁ Characteristics of traditional medicine and confusion of concepts
Attitudes	⦁ Respect and appreciation for cultural diversity, and avoidance of cultural destruction and ethnocentrism ⦁ Openness ⦁ Self-awareness, cultural awareness, and awareness of benefits
Subjective norms	⦁ Positive perception of traditional medicine, respect for patients, and avoidance of culturally unsafe behaviours ⦁ Biomedical model and evidence-based medicine ⦁ Acknowledge benefits of cultural safety and less ethnocentrism
Change intention	⦁ No subthemes
Agency	⦁ Able to accept cultural diversity in health care ⦁ Able to prevent culturally unsafe actions and improve the doctor-patient relationship
Discussion	⦁ No subthemes
Action	⦁ Better communication and relationship with patients and with other health professionals ⦁ Better outcomes for patients, physicians, and society ⦁ Dialogue and integration/balance/consensus between traditional medicine and modern medicine ⦁ Explore and investigate traditional medicine, and listen and learn from patients

### Conscious knowledge

Study participants told stories about learning the benefits of cultural safety training, such as increasing adherence of patients to treatment, generating a positive environment for health care, acquiring new knowledge and skills, and improving the doctor-patient relationship:

"[Cultural safety] helps to reach consensus between patients and me, it makes patients feel involved in their treatment without feeling diminished, therefore strengthening their adherence to medical treatment." (Participant 7)

“Taking the time to investigate the traditional practices of patients allows you to gain important knowledge and to develop a better relationship with patients.” (Participant 16)

Learned consequences of culturally unsafe behaviour included discriminating against or diminishing someone for their beliefs, assaulting the patient identity and culture, and barriers to approaching patients:

"Diminishing or discriminating against someone for their beliefs or for using traditional medicine is like attacking that person's identity and culture." (Participant 4)

Participants learned to recognize cultural diversity and the use of cultural practices in their setting, family, and even in themselves. They acknowledged traditional health practices as part of their cultural identity:

"In the lecture, we learned about remote tribes and communities, but I don't have to go that far to experience what I learned from the talk. Not only does my dad use traditional medicine, but most of my family, even me. For example, I drink a cinnamon infusion when I have a colic." (Participant 19)

Participants learned to differentiate between traditional health practices and alternative medicine, although the confusion remained for some students:

"I can now differentiate the concept of alternative medicine from that of traditional medicine. I understood that traditional medicine has a way of being and that it is part of someone else's culture." (Participant 4)

### Attitudes

This included respect and appreciation for cultural diversity and for their own culture:

"The most important thing is to recognize and respect cultural differences and protect them. Above all, preserve cultural practices and transmit them on from generation to generation, as this is part of the history of our region." (Participant 25)

Participants described an attitude of openness to recognize and accept the cultural differences that patients may have, thus preventing judgements and fostering learning from patients:

"[Cultural safety] training allows me to have an open mind to the beliefs and cultural practices that patients have; [It allows me to] take these aspects into account to prevent judgments and even to learn from them, from their experience" (Participant 7)

Participants reported awareness of their culture and biases, and acknowledged the ways their culture shapes clinical practice, often imposing their point of view:

"Now I understand that all of us, including the patients, grow with different customs, needs, and beliefs, and that all of us belong to a culture and therefore to a different way of seeing diseases and their treatment" (Participant 21)

### Subjective norms

Study participants reported a shift to a positive perception of traditional medicine, respect for patients, and willingness to address intercultural tensions:

"Now I see the impact that cultural practices have on patients' perception of health. I consider what people say about their beliefs, before I didn't even pay attention to it" (Participant 9)

"[Cultural safety] taught me that we should not belittle traditional medicine or believe that Western medicine is the truth and the solution to everything." (Participant 23)

Study participants acknowledged the impact that cultural practices have on patients' perception of health and reported less ethnocentrism:

"Because when we find a patient who has a culture with different beliefs from ours, we tend to ignore them; we believe that our culture is correct and that we are right, but [cultural safety training] makes us wonder if it is really like that."

They reported that while intercultural tensions were unpleasant before, they now see these tensions as opportunities to learn from the patient to provide better care:

"Before it was an unpleasant experience. There were many occasions in which the grandmothers talked about home remedies whose names were difficult for me to understand (I am not from this region); I doubted their effectiveness. Today, those experiences are not unpleasant anymore. I can now learn from the traditions of my patient, therefore providing better healthcare. " (Participant 17)

### Change intention

A study participant reported that cultural safety is what they want to do in their professional practice:

"I feel that this is what I want to do in my professional practice, to be able to help my patients, understand that traditional medicine is not wrong, and that [cultural safety] can enhance the doctor-patient relationship." (Participant 5)

### Agency

Some study participants feel that they can now accept cultural diversity in clinical practice and prevent culturally unsafe actions to improve the doctor-patient relationship.

"I am able to accept that there are diversity of beliefs and that each one has its cultural and scientific basis. It is a great contribution to our clinical practice." (Participant 26)

"I am able to improve the doctor-patient relationship. I can now create a relationship with patients from different cultures without imposing my thoughts, making judgments, or demeaning my patients and their families." (Participant 7)

### Discussion with peers

Trial participants discussed traditional and modern practices with patients, facilitating a space where both parties shared knowledge to inform decision-making. Additionally, they discussed the experience with nursing students who supported the cultural safety approach:

"I listened to the patients more; we discussed ways to take care of children, comparing things that they believed and had done with things that we knew from our medical knowledge; it helped to improve the connection with patients to ensure proper growth and development of babies. This was quite special because the nursing students who rotated with us also supported our approach, they learned from us." (Participant 1)

### Action

The students reported better communication and relationship with patients and with other health professionals, and better outcomes for patients, physicians, and society. This included patients feeling more understood, safe, and cared for, and increased knowledge of health professionals.

"Things that may seem so simple, but that for someone like the patient in the story, are important things. [Cultural safety] makes people feel safer, understood, and cared for, and they feel the desire to come back to see a doctor who also cares for them; in my opinion, it touches the most human part of medical practice." (Participant 15)

"Taking the time to investigate [traditional medicine] allows me to gain important knowledge and developing a better relationship with the patient." (Participant 16)

Participants reported efforts to find a balance or consensus between modern medicine and traditional health practices, based on dialogue with patients:

"I listened to the patients more; we discussed ways to take care of children, comparing things that they believed and had done with things that we knew from our medical knowledge. " (Participant 27)

"We were able to reach a consensus in which she understood that it is okay to go to the hospital in certain situations and I did not prevent her from continuing to use her traditional practices. Moreover, I learned about a home-made way of managing acute diarrhea” (Participant 7)

Both patients and study participants were able to share and learn from each other´s knowledge and practices to inform the health-decision making process. Some participants investigated traditional practices that they heard in clinical settings. This helped them to learn things that they do not learn at the faculty of medicine:

"During my gynecology and obstetrics rotation at Kennedy’s Hospital, it was common in prenatal check-ups to hear from several expectant mothers, talking about the use of *brevo* [medicinal plant] leaf baths. That was totally unknown to me at the time. I knew that *brevo* was the tree where the *brevas* grow [fruits that are traditionally eaten with *dulce de leche*]. I generally told the moms that the *brevo* baths were not necessary, that they were useless. However, while I was learning about cultural safety, I asked a patient the reason for these baths, to which she replied, ‘it is that they serve to be able to start contractions.’ After the consultation, I investigated the remedy and found that indeed the *brevo* leaf is used to start labor. Of course, I only knew of oxytocin and misoprostol, I had never heard of this practice, which as I read has been used since many years ago here in Colombia.” (Participant 20)

"when we asked the patients about traditional remedies, many told us about how drinking *hinojo* [fennel] infusion increase milk production." (Participant 9)

## Discussion

Our narrative assessment confirmed the positive impact of cultural safety training based on our co-designed curriculum [24] for Colombian medical students and interns. The most significant changes aligned with all seven elements of the CASCADA partial order of intermediate outcomes in behaviour change. The stories provided by trial participants highlighted changes in knowledge, attitudes, subjective norms, and action related to cultural safety.

Our findings fit with a review of cultural safety training changing student knowledge, attitude, self-confidence, and behaviour when interacting with Indigenous populations [8]. The review suggested that cultural safety training in medical education enhances respect for and acceptance of traditional and cultural health practices, it improves relationships between health professionals and patients from non-dominant cultures, and promotes healthier outcomes [8]. Another review reported changes in knowledge, attitudes, confidence, perceptions, collaboration, empathy, communication, behaviour, and practice [19]. A recent scoping review identified culturally safe strategies to improve rural Indigenous palliative care [34]. The authors described involvement of patients in decision making, self-reflection of care providers, and recognition of how culture shapes health care. A rapid review of diabetes care in Indigenous populations of Canada, Australia, New Zealand and the United States [35] identified positive effects of culturally safe interventions on clinical outcomes of patients, enhanced patient satisfaction and access to health care, and increased care provider confidence in providing care. These results support the idea of Arthur Kleinman that considering sociocultural aspects of care could enhance patient outcomes [36].

Our findings suggest that our training could encourage changes in practice of medical students and interns. While the literature reviews are entirely focused on Indigenous health, we provided evidence of the usefulness and relevance of cultural safety training among non-Indigenous populations that use traditional medicine, a widespread phenomenon in Latin American countries. Our results are relevant for medical educators interested in enhancing intercultural skills of medical trainees.

Our RCT explored the effectiveness of an innovative teaching strategy based on the transformative learning approach, which uses education that is interactive, participatory, and based on challenges [37]. Although we provided transformative learning through our game jam, the control group also received elements of transformative learning as this was requested by the directives at *La Sabana* University. Mezirow proposed transformative learning as a way to confront ethnocentrism [17]. Learners change beliefs about themselves, about others, and about practices, to make them more inclusive, open, and emotionally able to behavioural change [38]. Health-related students involved in transformative learning reported more confidence in caring for patients from non-dominant cultures [39–41]. Similarly, a recent game jam promoted self-discovery, reflections on identity, and support of the cultural identity of the Sami people in Finland [42]. Our results support the effectiveness of transformative learning for cultural safety training.

The reported areas of change in the stories reflected the content of our co-designed curriculum [24, 25] and included acknowledging culturally unsafe actions and their consequences, examining the students’ own attitudes, beliefs, and values, and how they shape their professional practice, willingness to listen and learn from their patients about traditional practices, and skills to discuss with patients to reach an agreement on their treatment, thus improving the doctor-patient relationship. Our results provide some evidence that cultural safety training based on our co-designed curriculum [24] may promote positive outcomes for both health professionals and patients.

We previously reported a positive perception of traditional and cultural health practices among Colombian medical students who participated in a five-month community-based cultural safety training program [13, 14]. The present study had a very substantial non-response rate, suggests positive outcomes for medical trainees after a much briefer (8 h) intervention. With the content overload in contemporary medical training and little time to include new subjects, our findings will be relevant to medical educators interested in cultural safety training.

### Limitations

A common limitation of medical education research based on self-reported data is social desirability bias [43], where participants feel pressured to report what they think the researcher wants to hear. We tried to reduce this bias by collecting anonymous data, making clear for the participants that their stories would not have any influence on their academic performance, and by suggesting that stories of negative changes or no change at all were also welcomed. There are reports of reduced desirability bias in web-based surveys, which we used in our study [44]. Despite this, we are cautious in interpreting our results. Only a self-selected minority of the students submitted a story, so this report emphasises stories from students interested in cultural safety. The stories describe how cultural safety training works *when it does work*, not the extent to which it worked.

To enhance trustworthiness of the data, the MSC stories were anonymized and we did not collect any additional information beyond responses to the MSC questions. A drawback of this approach was that we did not know if the students who reported their stories participated in the intervention or control group; both groups received cultural safety training. We did not know the sociodemographic characteristics of the subsample who provided the stories of change. Future research might consider changes reported by, for example, younger students and older students.

We are aware the narrative evaluation is at best an indirect assessment of change clinical practice. Future cultural safety research could use more objective measures, like direct observation of trainees in clinical settings and patient-related outcomes, ideally measured on or reported directly by patients.

## Conclusion

The stories of change provided insights into the likely nature of impact of cultural safety education on a results chain from conscious knowledge to action after an eight-hour transformative learning training session. Our results support the idea that cultural safety training based on our co-designed curriculum [24] can produce positive outcomes for both health professionals and patients. We encourage medical educators to conduct and report other cultural safety training experiences, ideally using patient-related outcomes or direct observation of medical trainees in clinical practice.

### Protocol

The protocol of this RCT was accepted for publication before completion of recruitment [15].

## Supplementary Information

**Additional file 1. **RCT protocol.

**Additional file 2. **Standards for Reporting Qualitative Research checklist.

**Additional file 3. **Deductive thematic analysis results - complete list of quotes.

## Data Availability

The datasets used and/or analysed during the current study will be available from the corresponding author on reasonable request.

## Abbreviations

RCT: Randomised Controlled Trial
MSC: Most Significant Change technique
CASCADA: Conscious knowledge, Attitudes, Subjective norms, Change intention, sense of Agency, socialization/Discussion, and behavior change/Action

## References

[1] World Health Organization. WHO Traditional Medicine Strategy 2002–2005. 2002;:1–74. https://apps.who.int/medicinedocs/en/d/Js2297e/. Accessed 3 Aug 2020.

[2] World Health Organization (WHO). Traditional, Complementary and Integrative Medicine. Health topics. 2022. https://www.who.int/health-topics/traditional-complementary-and-integrative-medicine#tab=tab_1.

[3] Parra L, Pacheco AM (2006). Monologue or Intercultural Dialogue Between Medical Systems? An Educational Challenge for Medical Sciences. Rev Cienc Salud.

[4] Bernal R, Cárdenas M, Giuffrida A (2007). Race and Ethnic Inequality in Health and Health Care in Colombia. Racial and Ethnic Disparities in Health in Latin America And The Caribbean.

[5] Horvat L, Horey D, Romios P, Kis-Rigo J (2014). Cultural competence education for health professionals. Cochrane Database Syst Rev..

[6] Beach MC, Price EG, Gary TL, Robinson KA, Gozu A, Palacio A (2005). Cultural Competence. Med Care.

[7] Lie DA, Lee-Rey E, Gomez A, Bereknyei S, Braddock CH (2011). Does Cultural Competency Training of Health Professionals Improve Patient Outcomes? A Systematic Review and Proposed Algorithm for Future Research. J Gen Intern Med.

[8] Kurtz DLM, Janke R, Vinek J, Wells T, Hutchinson P, Froste A (2018). Health Sciences cultural safety education in Australia, Canada, New Zealand, and the United States: a literature review. Int J Med Educ.

[9] Williams R (1999). Cultural safety - what does it mean for our work practice?. Aust N Z J Public Health.

[10] Blanchet Garneau A, Pepin J. La sécurité culturelle : une analyse du concept. Rech Soins Infirm. 2012;N° 111:22. 23409542

[11] Kirmayer LJ (2012). Rethinking cultural competence. Transcult Psychiatry.

[12] Pon G (2009). Cultural Competency as New Racism: An Ontology of Forgetting. J Progress Hum Serv.

[13] Pimentel J, Sarmiento I, Zuluaga G, Andersson N (2020). What motivates medical students to learn about traditional medicine? A qualitative study of cultural safety in Colombia. Int J Med Educ.

[14] Pimentel J, Kairuz C, Merchán C, Vesga D, Correal C, Zuluaga G (2021). The Experience of Colombian Medical Students in a Pilot Cultural Safety Training Program: A Qualitative Study Using the Most Significant Change Technique. Teach Learn Med.

[15] Pimentel J, Cockcroft A, Andersson N (2020). Impact of Co-Designed Game Learning on Cultural Safety in Colombian Medical Education: Protocol for a Randomized Controlled Trial. JMIR Res Protoc.

[16] Pimentel J, Cockcroft A, Andersson N (2021). Impact of game jam learning about cultural safety in Colombian medical education: a randomised controlled trial. BMC Med Educ.

[17] Mezirow J (1997). Transformative Learning: Theory to Practice. New Dir Adult Contin Educ.

[18] Davies R, Dart J (2005). The ‘Most Significant Change’ (MSC) Technique.

[19] Medel S. The Impact of Indigenous Cultural-Safety Education Programs: A Literature Review. Burnaby (British Columbia): Simon Fraser University; 2019.

[20] O’Brien BC, Harris IB, Beckman TJ, Reed DA, Cook DA (2014). Standards for Reporting Qualitative Research. Acad Med.

[21] La Sabana University. The University in Figures. 2018. https://www.unisabana.edu.co/nosotros/la-sabana-en-cifras/. Accessed 1 Apr 2020.

[22] Taylor EW (2007). An update of transformative learning theory: a critical review of the empirical research (1999–2005). Int J Lifelong Educ.

[23] Preston J (2012). a, Chastine J, O’Donnell C, Tseng T, MacIntyre B. Game Jams Int J Game-Based Learn.

[24] Pimentel J, Kairuz C, Suárez L, Cañón A, Isaza A, Zuluaga G (2022). A co-designed curriculum for cultural safety training of Colombian health professionals: sequential-consensual qualitative study. Can Med Educ J..

[25] Pimentel J, Zuluaga G, Isaza A, Molina A, Cockcroft A, Andersson N, Costa AP, Reis LP, Moreira A (2019). Curriculum Co-design for Cultural Safety Training of Medical Students in Colombia: Protocol for a Qualitative Study. Computer Supported Qualitative Research.

[26] Braun V, Clarke V (2006). Using thematic analysis in psychology. Qual Res Psychol.

[27] Prochaska JO. Transtheoretical Model of Behavior Change. In: Encyclopedia of Behavioral Medicine. Springer, Cham; 2020. p. 2266–70.

[28] Valente TW, Paredes P, Poppe PR (1998). Matching the Message to the Process The Relative Ordering of Knowledge, Attitudes, and Practices in Behavior Change Research. Hum Commun Res.

[29] Andersson N, Beauchamp M, Nava-Aguilera E, Paredes-Solís S, Šajna M (2017). The women made it work: fuzzy transitive closure of the results chain in a dengue prevention trial in Mexico. BMC Public Health.

[30] Albarracín D, Johnson BT, Fishbein M, Muellerleile PA (2001). Theories of reasoned action and planned behavior as models of condom use: A meta-analysis. Psychol Bull.

[31] Ajzen I (1991). The theory of planned behavior. Organ Behav Hum Decis Process.

[32] Patton MQ (1999). Enhancing the quality and credibility of qualitative analysis. Health Serv Res.

[33] Shenton AK (2004). Strategies for ensuring trustworthiness in qualitative research projects. Educ Inf.

[34] Schill K, Caxaj S (2019). Cultural safety strategies for rural Indigenous palliative care: a scoping review. BMC Palliat Care.

[35] Tremblay M-C, Graham J, Porgo TV, Dogba MJ, Paquette J-S, Careau E, et al. Improving Cultural Safety of Diabetes Care in Indigenous Populations of Canada, Australia, New Zealand and the United States: A Systematic Rapid Review. Can J Diabetes. 2019. 10.1016/j.jcjd.2019.11.006 32029402

[36] Katon W, Kleinman A. Doctor-Patient Negotiation and Other Social Science Strategies in Patient Care. In: The Relevance of Social Science for Medicine. Springer Netherlands; 1981. p. 253–79.

[37] Kitchenham A (2008). The Evolution of John Mezirow’s Transformative Learning Theory. J Transform Educ.

[38] Van Schalkwyk SC, Hafler J, Brewer TF, Maley MA, Margolis C, McNamee L (2019). Transformative learning as pedagogy for the health professions: a scoping review. Med Educ.

[39] Riner ME (2013). Globally Engaged Nursing Education with Local Immigrant Populations. Public Health Nurs.

[40] Smith-Stoner M, Hand MW (2008). A Criminal Trial Simulation Nurse Educ.

[41] Briscoe L (2013). Becoming culturally sensitive: A painful process?. Midwifery.

[42] Laiti O, Harrer S, Uusiautti S, Kultima A (2021). Sustaining intangible heritage through video game storytelling - the case of the Sami Game Jam. Int J Herit Stud..

[43] Gozu A, Beach MC, Price EG, Gary TL, Robinson K, Palacio A (2007). Self-Administered Instruments to Measure Cultural Competence of Health Professionals: A Systematic Review. Teach Learn Med.

[44] Kreuter F, Presser S, Tourangeau R (2008). Social desirability bias in CATI, IVR, and web surveys: The effects of mode and question sensitivity. Public Opin Q.

