# Supplementary material for: The most significant change for Colombian medical trainees going transformative learning on cultural safety: qualitative results from a randomised controlled trial
Source: BMC Med Educ. 2022 Sep 10;22:670. doi: 10.1186/s12909-022-03711-1 (PMC9463722; doi:10.1186/s12909-022-03711-1)
Supplement: Supplementary file 1 — Additional file 1. RCT protocol. [file 12909_2022_3711_MOESM1_ESM.docx]

**Additional file 1. RCT protocol**

# Impact of Co-Designed Game Learning on Cultural Safety in Colombian Medical Education: Protocol for a Randomized Controlled Trial

Juan Pimentel ^1,2,3^, MD, MSc, PhD(c); Anne Cockcroft ^1,4^ MBBS, MD; Neil Andersson ^1,4^ PhD, MD

^1^ CIET/PRAM, Department of Family Medicine, McGill University, Montreal, QC, Canada

^2^ Facultad de Medicina, Universidad de La Sabana, Chía, Colombia

^3^ Escuela de Medicina y Ciencias de la Salud, Universidad del Rosario, Bogotá, Colombia

^4^ Centro de Investigación de Enfermedades Tropicales (CIET), Universidad Autónoma de Guerrero, Acapulco, Guerrero, Mexico.

**Note:** Our study protocol has been peer-reviewed and it was published on JMIR Research Protocols:

Pimentel J, Cockcroft A, Andersson N. Impact of Co-Designed Game Learning on Cultural Safety in Colombian Medical Education: Protocol for a Randomized Controlled Trial. JMIR Res Protoc. 2020;9:e17297.

The protocol is available: <https://www.researchprotocols.org/2020/8/e17297/pdf>

## Abstract

**Background:** Cultural safety encourages practitioners to examine how their own culture shapes their clinical practice, and to respect their patients’ worldviews. Lack of cultural safety in health care is linked to stigma and discrimination towards culturally diverse patients. Training in cultural safety has considerable challenges. It is an unappealing subject for medical students and requires behavioural changes in their clinical practice. Game jams, collaborative workshops to create and play games, have recently shown effectiveness and engaging potential in university-level education.

**Objective:** The trial aims to determine whether medical student participation in a game jam to design an educational game on cultural safety is more effective than a standard lesson on cultural safety in terms of change in students’ self-reported intended patient-oriented behavior.

**Methods:** A parallel group, two arm, randomised controlled trial with a 1:1 allocation ratio will randomize 340 medical students and 60 medical interns (n=400) at the Faculty of Medicine at *La Sabana* University, Colombia (170 students and 30 medical interns to each arm). The intervention group will participate in an 8-hour game jam comprised of: (i) a preliminary lecture on cultural safety and game design; (ii) a game building session where groups of students will create educational games about cultural safety; and (iii) a play-test session in which students will play and learn from each other’s games. The control group will receive a standard lesson, including a 2-hour lecture on cultural safety, followed by a 6-hour workshop to create posters about cultural safety. Online self-administered 30-item Likert-type questionnaires will assess cultural safety self-reported intended behavior before, immediately after, and six months after the intervention. An intention-to-treat approach will use a t-test including 95% confidence intervals to determine the significance of the effect of the intervention, including within- and between-group comparisons. The qualitative Most Significant Change technique will explore the impact of the intervention on the clinical experience of the students.

**Results:** Study enrollment began in July 2019. 640 students completed the baseline survey and were randomised. Data collection is expected to be complete by July 2020 and results are expected in October 2020.

**Conclusions:** The research will develop participatory methods in game-based learning co-design that might be of relevance to other subjects. Ultimately, it should foster improved cultural safety skills for medical students, improve quality of health services for diverse cultural groups, and contribute to enhanced population health. Game learning may provide an innovative solution to a long-standing and neglected problem in medical education, helping to meet the educational expectations and needs of millennial medical students.

**Trial registration:** ISRCTN registry. The trial registration number is ISRCTN14261595 <http://www.isrctn.com/ISRCTN14261595>

**Keywords:** transformative learning, medical education, cultural safety, participatory research, game jam

## Introduction

### Cultural safety training

Although cultural safety is an evolving term and lacks a formal definition,[1] it is often described as a space “that is spiritually, socially, emotionally and physically safe for people; where there is no assault, challenge or denial of their identity, of who they are, and what they need.”(p272)[2] The concept originated in New Zealand to address the disconnect between the type of health care that Indigenous Maori people were receiving and the culturally-congruent care that they were advocating for.[3]

Cultural safety has gradually gained attention because it offers a more comprehensive and respectful way to approach culture, in many settings replacing the current standard, which is cultural competence.[4] Cultural safety is distinct from cultural competence, in that it invites culturally diverse patients and their communities to co-design and evaluate culturally safe health care.[1][5] Recent evidence suggests that cultural safety education is linked to better relationships between culturally diverse patients and clinicians and improved health outcomes.[2]

The Royal College of Physicians and Surgeons of Canada will soon require all medical residency programs to provide mandatory cultural safety training.[6,7] There is, however, little research on how to implement this approach in medical education,[8] and how health professionals acquire cultural safety skills is poorly understood.[9]

There are additional challenges to promoting cultural safety in medical education. Educators might find cultural safety complicated to teach and medical students may perceive it dull.[10]^,^ Contemporary medical training is almost everywhere overloaded, with little space to include an entirely new if very important subject. Millennial medical students – the birth cohort between 1979 and 2000 [11] – have new learning relationships with technology, creativity, and amusement that modern teaching strategies cannot overlook.[12] Finally, cultural safety training goes beyond merely knowledge acquisition; it must promote a *transformative* experience to impact students’ behavior in clinical practice. The theory of transformative learning provides a framework to address these challenges.[13]

### Transformative learning and game co-design

Mezirow describes transformative learning as a process that changes frames of reference, “the structures of assumptions through which we understand our experiences.”(p5)[13] Frames of reference are comprised of habits of mind, which are habitual ways of thinking and acting, and points of view, which are beliefs, values, and attitudes.

Mezirow argues that ethnocentrism, defined as “the predisposition to regard others outside one’s own group as inferior,”(p6)[13] is an example of a habit of mind. Ramsden, the Maori nurse who developed the concept of cultural safety, proposes that confronting ethnocentrism must be the first step in cultural safety training.[3] Transformative learning may be, therefore, suitable to provide cultural safety training to medical students.

Transforming frames of reference requires reflection on the assumptions upon which learners base their habits of mind and points of view.[13] In transformative learning, people become critically reflective of their assumptions through education that is participatory and interactive and through group problem-solving or communicative learning.[14]

*Game jams* provide an environment to foster learning through interacting and communicating with others,[15] an essential aspect of transformative learning. These participatory events allow attendees to create games (digital or board games) in a time-constrained environment.[16] Unlike other educational approaches, game jams could offer a solution to the challenges of cultural safety in medical education by (i) engaging Millennial students through a culture of creativity and learning, play testing, and idea sharing; (ii) supporting a transformative process of learning-by-doing while enhancing creative thinking, problem-solving, communication, and innovation; and (iii) promoting transformative learning in less time, thus offering an alternative to overloaded medical curricula.

Fowler and collaborators recently found that game jam participation could improve computing students' performance.[15] We are not aware, however, of any reported experience using game jams to train medical students. Our primary objective is to determine whether medical student participation in a game jam to design an educational game on cultural safety is more effective than a standard lesson on cultural safety in terms of change in students’ self-reported intended patient-oriented behavior. Our secondary objectives are to: (i) determine the impact of the intervention (game jam) compared with the control (standard lesson on cultural safety) on students’ confidence in their general transcultural skills; and (ii) assess the impact of participation in the game jam through a narrative approach that identifies in their own words the effect of the learning on cultural safety in their clinical practice.

## Methods

### Trial design

A parallel group, two arm randomised controlled trial (RCT) with 1:1 allocation will compare participation in a game jam with a standard lesson on cultural safety. The RCT will answer the question: *Among medical students and interns from La Sabana University, does participating in a game jam for cultural safety training, in comparison to a standard lesson on cultural safety, result in increased change in students and interns’: (a) self-reported intended behavior; (b) confidence in general transcultural skills; and (c) reported change in clinical practice?* Table 1 presents the PICOT components of the research question. This protocol description follows the SPIRIT (Standard Protocol Items: Recommendations for Interventional Trials) 2013 statement[17] (Multimedia Appendix 1: SPIRIT checklist of the study protocol).

Table 1. Population, intervention, contrast, outcome, and timing (PICOT) of the RCT

| **Components** | **Description** |
| --- | --- |
| Population | Undergraduate medical students and medical interns at *La Sabana* University in Colombia |
| Intervention | Game jam aimed at fostering cultural safety in clinical practice |
| Contrast | Standard lecture and workshop on cultural safety |
| Outcome | (i) Cultural safety intended patient-oriented behavior change outcomes from knowledge to action; (ii) Students’ confidence in general transcultural skills; and (iii) Qualitative understanding of the change experienced by participants in their clinical practice |
| Timing | Before the intervention, immediately following the teaching session, and six months post-intervention |

### Study setting

We will conduct the RCT at the Faculty of Medicine at *La Sabana* University in the municipality of Chía, Colombia. Chía is a small town located 15 km from Bogotá, the capital of Colombia. *La Sabana* University is a private higher education institution that has 8,926 undergraduate students; 22% of these students come from a low socioeconomic level, 52% belong to the middle class, and the remaining 26% come from higher socioeconomic backgrounds.[18] Presently, there are 956 students enrolled in medical school and 256 medical interns (n=1,212).[18] At *La Sabana*, the duration of the MD program is seven years. As part of that training, all medical students must undergo a one-and-a-half-year medical internship before graduating.

### Eligibility criteria

The inclusion criteria are: (i) being a medical student or medical intern at any level of training and (ii) giving informed consent. The exclusion criterion is not wanting to participate in the study.

### Interventions

#### Game jam

The intervention will consist of a game jam aimed at creating a low-tech prototype of an educational game to foster cultural safety in medical education. Groups of five or six students or medical interns will create an educational game prototype from scratch. We will follow a six-step game jam protocol based on Macklin’s *Planning your game jam* guidelines[19] (Figure 1).

**
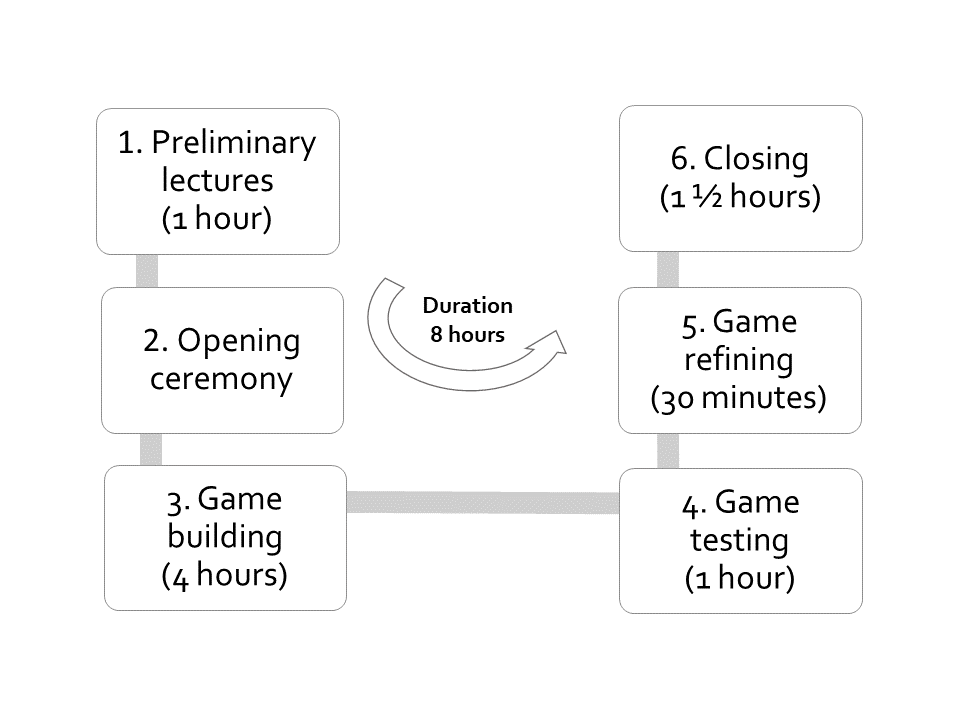
Figure 1. Game jam protocol**

1) Preliminary lecture session (one hour): this is comprised of:

(i) A 30 minute lecture on cultural safety will follow a cultural safety curriculum co-designed with local community members knowledgeable about cultural and traditional health practices[20]; and (ii) a 30 minute lecture on game design.

2) Opening ceremony: game jams usually start with opening comments from the host. We will welcome the participants and share the agenda and rules of the game jam.

3) Game building (four hours)**:** this is comprised of six steps:

a) We will invite participants each to write a brief narrative of when they witnessed (or heard of) discrimination or disrespect against a patient because of their traditional health practices, and the consequences of this discrimination.

b) Participants will share their brief stories within their game jam group to discuss and select the story (based on consensus) that best describes discrimination or disrespect against a patient. A key component of this step is to imagine/brainstorm the fullest range of possible consequences – from trivial to life-threatening.

c) Participants will anonymise the selected story as that of a fictional medical student who has to do a primary care clinical rotation in a local community where she/he faces intercultural tensions in clinical practice. The participants will then convert this narrative into a game, and will define a set of rules, rewards, and penalties.

d) Participants will discuss the factors that hypothetically led the medical student to be discriminatory or disrespectful towards their patient in the story. After the discussion, each group will select and integrate 5-10 factors into the game. The *challenge* here is that players have to become aware gradually that these factors can lead to disrespect or discrimination against culturally diverse patients as they play the game. Concretely, the jammers will be expected to add factors such as the hegemony of evidence-based medicine, colonization and ethnocentrism, and other factors defined in the co-designed cultural safety curriculum.

e) Participants will discuss what can be done to address each of the selected factors that contributed to the disrespect or discrimination experienced by the patient in the narrative. Each group will select and integrate 5-10 actions to promote dialogue and respect towards culturally diverse patients in clinical encounters into their game. The *challenge* is that players learn to respect patients who use traditional health practices in clinical encounters as they play the game.

f) The students will discuss and identify ideas to start working with the patient as a team in the health care decision-making process. This involves engaging in dialogue with the patient to invite them to bring their cultural and traditional practices to inform the health care decision-making process. The traditional practices will be pre-defined by our co-designed curriculum.[20] The *challenge* is that players of the game have to learn how to work with traditional medicine users to jointly make health decisions that are culturally safe.

4) Game testing (one hour)**:** groups will learn from each other’s solutions, ideas, and resources, thus strengthening the cultural safety learning process. At least one member of each group will stay at their workplace to present their game. The remaining students of the group will rotate to play the games created by other groups, thus ensuring that participants from all groups will play at least two additional games. Before the end of the session and using Google Forms, we will ask the students to evaluate other groups’ games in different categories aligned with each of the challenges.

5) Game refining (30 minutes)**:** after playing and testing other teams’ games, each group will have new ideas for refining their own game. Groups will then return to their workplace and apply lessons to improve their own game. Each group will fill a form to register their game on Google Forms.

6) Closing (one-and-a-half hour): we will bring the full group together for final presentation of the games. Each group will have to provide a brief description of their game and to discuss how they solved each of the game building challenges. We will facilitate this session to highlight the underlying concepts of cultural safety. Finally, we will award prizes in three different categories aligned with each of the challenges.

#### Control

The control group will receive a one-and-a-half-hour lecture on cultural safety in medical education by an expert in cultural safety. The lecture will be a standard lesson using PowerPoint slides and will cover the same key concepts used in the game jam, including (a) definition of cultural safety; (b) consequences of cultural tensions in health care; (c) self-awareness; (d) Colombian cultural health practices; and (e) respect for culturally diverse patients. The lecture will be based on our co-designed curriculum.[25] The session will be followed by a 15-minute period to make comments and to ask questions and a 15-minute break.

After the break, the students will participate in a six-hour workshop based on cultural safety selected readings. Groups of five or six students or medical interns will answer ten open-ended questions based on the lecture and the readings, and will create a poster to graphically display their responses to other students. Similar to the game jam session, we will split each group and encourage a rotation process where participants from all groups will learn from at least two additional posters. Before the end of the session and using Google Forms, we will ask the students to evaluate other groups’ posters in different four categories: creativity, coverage of the topic, graphics and pictures, and layout and design.

In the closing session, the best groups will present their posters to the group at large. In this session, we will unpack and highlight the key concepts of cultural safety. Similar to the game jam session, we will award prizes in the four evaluated categories. Matching that in the intervention, the duration of participation in the control group will be 8 hours.

### Criteria for discontinuing or modifying allocated interventions

Participants are free to withdraw from the trial at any point. We will collect reasons for withdrawal from subjects who drop out of the trial.

Participants will not be able to switch groups once they have been randomised to the intervention or control arms, even if they request to do so. Using participants’ lists, the facilitators will ensure that participants remain in their designated groups.

### Strategies to improve adherence to intervention

We will recruit ten to twenty game jam facilitators to support participants and to ensure all groups are able to meet the challenge of each step of the game jam protocol. The facilitators will be final-year medical students or medical interns interested in cultural safety research or game-based learning. We will train the facilitators during one month before the game jam to ensure that they will have the skills successfully to support the game jam participants in their learning process.

We will record the attendance to the intervention and control arms activities. Along with the names of the participants, we will record the date, hour, and their signatures.

### Relevant concomitant care and interventions that are permitted or prohibited during the trial

Contamination is a concern of parallel-group RCTs in education. This occurs when individuals that are receiving the intervention “leak information” that influences results in the control group. This usually reduces the measured intervention contrast, making it more difficult to find a significant difference between groups.[21]

In this study, we cannot guarantee that contamination will not occur, but we will attempt to minimize this risk. We will ask the students to avoid real-time communication with their peers (for example using their cell phones) and will conduct intervention and control activities simultaneously, with the two groups in auditoriums in different buildings. Both groups will have different lunch times.

### Outcomes

Primary outcome: our primary outcome will be students’ self-reported intended patient-oriented behavior. Our outcome is self-reported because it uses students’ opinion regarding their perceived change; intended, because we are assessing students’ planned behavior instead of actual practice/action; and patient-oriented because we measure responses to clinical scenarios indicating intended behavior change. We will assess intended behavior change from the baseline to immediately following the teaching session, from immediately following the teaching session to six months post-intervention, and from the baseline to six months post-intervention.

We will use the CASCADA model of planned behavior to inform our primary outcomes.[22] The model includes the following variables: **C**onscious knowledge was the response to the statement: Considering the cultural beliefs of my patients is not important for health-decision making. **A**ttitude to cultural safety was derived from the statement: It is not worth considering the cultural beliefs of my patients to improve their health. **S**ubjective norm used the statement: Although many physicians disapprove cultural beliefs, I think that these beliefs could improve my patients’ health. Intention to **C**hange derived from the statement: I do not think that learning about cultural beliefs is necessary to provide good health care to my patients. **A**gency was the response to the statement: I feel prepared with the knowledge and skills to prudently incorporate my patients' cultural practices in the health-decision making process. **D**iscussion derived from the response to the statement: I will discuss cultural safety with other students and physicians so they can prudently incorporate their patients' cultural practices in the health-decision making process. Agency and Discussion replace perceived behavior in a conventional theory of planned behavior.[23] Agency involves both self-efficacy and collective efficacy. The CASCADA model includes Discussion as an additional factor that facilitates behavior change.[24] **A**ction was the response to the statement: I will never be open to include my patients’ cultural beliefs and practices in the health-decision making process. Participatory research experts have successfully used the CASCADA model to explore dengue prevention behavior in previous trials.[24]

Secondary outcomes: (i) students’ confidence (transcultural self-efficacy) in their general transcultural skills; and (ii) qualitative understanding of the impact of the intervention in the clinical practice of medical students and medical interns through the Most Significant Change Technique. We will assess transcultural self-efficacy in the baseline, immediately following the teaching session, and six months post-intervention, and we will conduct the qualitative assessment in both groups six months after the intervention.

Output: each student group of the intervention arm will create a co-designed low-tech prototype of a serious game to foster cultural safety in medical students. Some of these prototypes may serve as blueprints for future fully developed games, or as input for future educational videogames.

In addition to the quantitative outcomes of the RCT, we will use the qualitative Most Significant Change narrative technique[25] to collect and analyze stories of change from the medical students six months after the intervention. This technique will allow us to capture meaningful changes in the students’ clinical practice, which may not be apparent from the quantitative evaluation.

### Participant timeline

Figure 2 shows the CONSORT flow diagram of the RCT.[26]

**Figure 2. CONSORT flow diagram of the RCT**

**
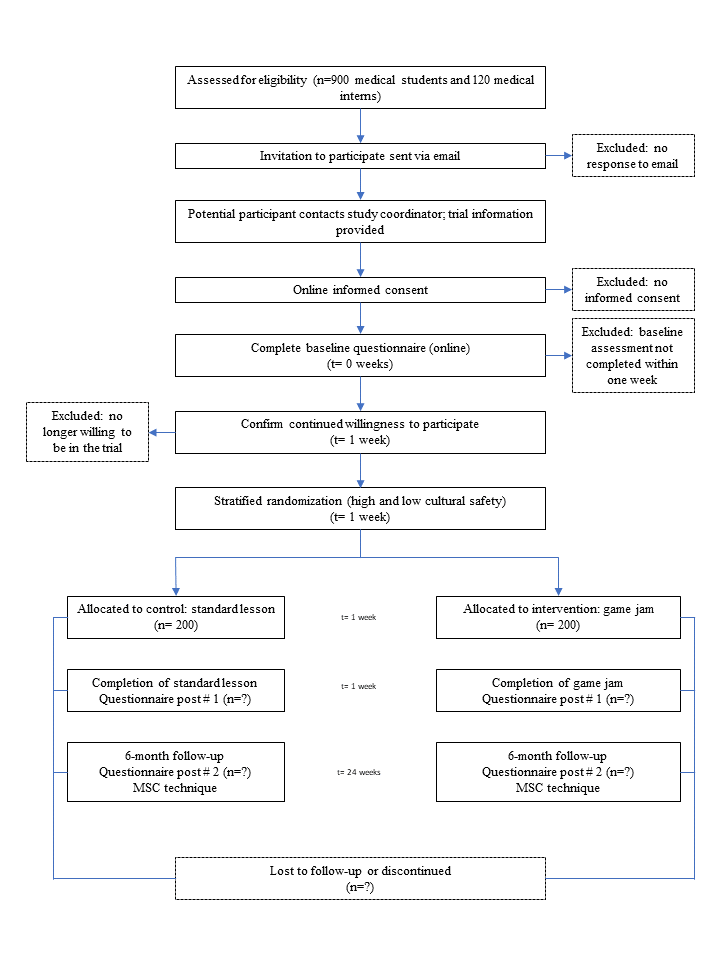
**

### Sample size

Using preliminary results from our pilot RCT, we found an effect size (Cohen’s *d*) of 0.25 between intervention and control arms after the teaching session (mean in game jam group = 26.9 SD 4; mean in control group = 25.9 SD 4). We calculated the sample size using the *pwr* package in R.[27] We expect that a group size of 199 participants in the game jam group and 199 participants in the control group (sample size = 398) will allow to detect an effect size of (Cohen’s d) 0.25, with a two-sided alpha = 0.05 and a power = 0.8 (see Figure 3). Since we observed considerable contamination in the pilot RCT, an effect size of 0.25 is conservative.

**
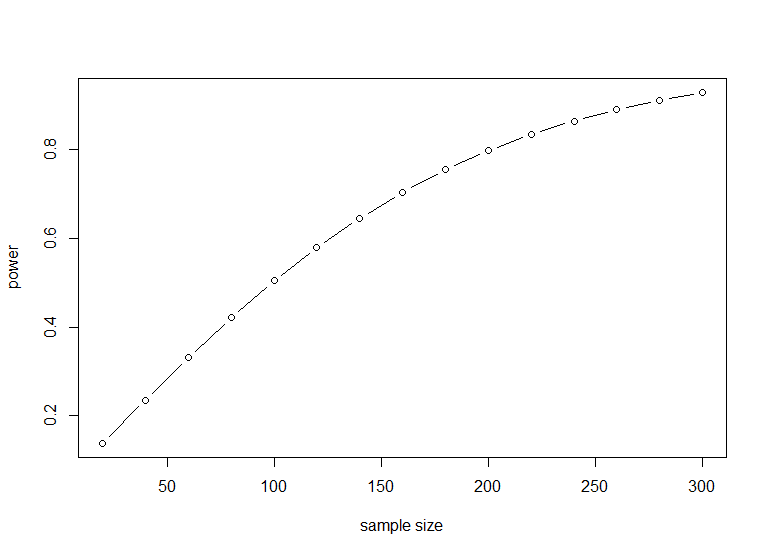
Figure 3. One-arm power curve for sample size calculation**

### Recruitment

We will contact the medical students and medical interns using *La Sabana* University’s mailing lists and will e-mail invitations for voluntary participation in the project. For those willing to participate, we will send further information about the project as well as the date and place of the intervention. We will ask interested students to complete the online informed consent and baseline questionnaire one week before the RCT.

### Allocation

A potential source of bias in our study is a possible imbalance in the level of cultural safety training between the intervention and control groups before the intervention. The reason for this issue is that in Colombia around 40% of the population uses cultural and traditional practices to maintain their health.[28] Therefore, some students will be familiar with traditional health practices, probably making them more likely to embrace the cultural safety approach compared with students not familiar with these practices.

To address this potential bias, we will use stratified randomization by cultural safety score at baseline. Based on the preliminary results of the baseline survey, we will split the group of medical students into two groups: low and high level of cultural safety knowledge. Computerized randomization will allocate the students either to the intervention or control arm and we will use equal allocation between treatment arms. The study coordinator will be responsible for generating the allocation sequence, enrol participants, and assign participants to interventions.

### Data collection methods

Data collection: we will collect quantitative data at three time-points: baseline, immediately after the intervention, and six months after the intervention, and will collect the narratives of change only after six months of the game jam. Participants will enter quantitative data using mobile devices and SurveyMonkey self-administered questionnaires. Similarly, they will upload their stories of change using a pre-designed format on Google forms. We report our online instruments in accordance with the Checklist for Reporting Results of Internet E-Surveys -CHERRIES-[29] (Multimedia Appendix 2).

Instrument and quantitative data to be collected: to the best of our knowledge, there are no validated research instruments to measure cultural safety outcomes in healthcare providers. A recent systematic review[30] exploring instruments to assess cultural competence (and aligned concepts) identified ten instruments. All of them were self-administered and based on respondent perceptions. Most of these instruments (5/10) measured cultural competence; none were designed to measure cultural safety.

Our recently published scoping review identified that the Transcultural Self–Efficacy Tool — Multidisciplinary Healthcare Provider version (TSET–MHP) has been used to assess the effectiveness of game-based learning interventions to promote cultural competence.[31] Researchers report a growing body of evidence supporting validity and reliability of the instrument.[32] The instrument assesses cognitive, practical, and affective learning dimensions that can be categorized within the classic knowledge, attitudes, and skills behavior change outcomes.

Brascoupé points out that cultural competence provides a foundation for cultural safety.[33] Ramsden sees cultural safety training as a dynamic process moving from cultural awareness to cultural competence to cultural safety.[3] Following this rationale, we will use a 30-item instrument comprised of three parts. The first part (five items) will explore sociodemographic factors of the students. This includes sex, age, level of training, place of birth, socioeconomic status, and traditional health practices used in the family. The second part (15 items) will be based on the Likert-type TSET-MHP and will explore knowledge, attitudes, skills, and behaviors of cultural competence.

For the third part of the instrument (cultural safety), we developed a Likert-type preliminary version based on our CASCADA variables (see primary outcome) and piloted it for validity and reliability in our pilot RCT.

Validity and reliability: using data from our pilot RCT, we followed the process proposed by Jeffreys[34] to improve the validity and reliability of the third part of our instrument. In the pilot study, the questionnaire included the open question: *how can we improve this instrument?* An inductive thematic analysis[35] of responses identified suggestions to adjust our survey. We shared the adjusted version of the instrument by email with two general practitioners, one medical intern, six medical students, and four cultural safety experts. We adjusted the instrument according to their comments and agreed on the content validity of the instrument by consensus.

To increase the construct validity of our instrument, we used the contrasted group approach, which explores the difference between two separate groups.[34] To increase the predictive validity of our instrument, we looked at the score difference between two timeponts.[36] Reliability explores the degree of accuracy and consistency in measurement. Using R Studio v1.1.419, we calculated Cronbach’s alpha[37] to determine the internal consistency of our instrument. Since our instrument was short (<10 items), we expected a value of >0.5.[38] We complemented the reliability exploration using the test-retest method to explore the stability of the instrument.[34] We report the validity and reliability results of our instrument in the pilot RCT, which is not yet published.

Qualitative data to be collected: to explore students’ stories of change after cultural safety training, we will use the Most Significant Change approach, which is a narrative technique that allows participants to communicate changes most meaningful to them.[39] Using a pre-defined format in Google forms, we will ask participants to write down and enter their stories based on the following questions: *(a) what do you believe was the most significant change in your clinical practice as a result of your participation in the activity [game jam or standard lesson] 6 months ago? (b) can you please share a real-life story depicting this change? (c) why do you think this story is significant?*

The instructions will make clear that participants should feel free to write down stories of negative changes or to say that they did not experience any change at all. Only medical students involved in clinical practice and medical interns (third to seventh year of medical school) will be invited to participate in this part of the RCT.

Methods to maximise completeness and quality of data: The study coordinator and facilitators will be physically present while collecting the data at each time point to ensure the completeness of data. Additionally, we will use several validation options to increase the quality of the data: specific number range, specific character range, date validation, email address format, and prompts that alert participants when they enter incomplete or invalid answers.

In this study, the familiarity of millennial medical students with technology and computer-based education supports using Web-based questionnaires, which reputedly decrease social desirability bias.[40] Assured of anonymity, respondents should be less concerned about what others may think about their responses, including peers and professors.[41] Data reliability in Web-based questionnaires is reportedly equal to or better than traditional paper-based approaches.[42] Examples include data on self-reported perceived health status, oral contraceptive use, and smoking and alcohol use. Web-based questionnaires are also faster to complete and typically cheaper than traditional approaches, making them ideal for our research.

Methods for ensuring secure storage of data: SurveyMonkey and Google responses are stored in a worksheet that can only be accessed through an account login. Data transmission uses Secure Sockets Layer to encrypt information during transport. The data storage is as secure as most other systems that store survey information. After downloading the data, we will delete it from SurveyMonkey and Google Forms. We will be responsible for ensuring the data are securely stored for seven years and then destroyed in accordance with CIET guidelines for security, storage, and eventual destruction of data records.[43]

### Methods for analyzing data

Primary analysis***:*** using an intention-to-treat approach, we will perform a t-test including 95% confidence intervals to determine the effect of the intervention, including within-group comparisons (baseline and post-intervention I and II) and between-group comparisons (treated versus control post-intervention).

Secondary analysis: we will adjust for sociodemographic baseline variables. We will conduct both a logistic regression and the Mantel-Haenszel procedure, which will allow comparing the results using two different statistical approaches based on different assumptions. Contrary to logistic regression, the Mantel-Haenszel procedure is nonparametric, which makes it free of any statistical assumption and therefore more robust.[44]

Supplementary analysis: both intervention and control arms will engage in group work during the training session. We will use generalised linear mixed modelling (GLMM), paying special attention to a possible cluster enhancement as participants in some study groups generate stronger learning dynamics than others. Additionally, we will follow the methods proposed by Andersson and collaborators[24] to conduct probabilistic transitive closure of each CASCADA result chain, which will allow us to explore walks and blocks between intermediate outcomes and to obtain the cumulative net influence. Probabilistic transitive closure is usually conducted on fuzzy cognitive maps where the weights of relationships between concepts are interpreted as probabilities.[45] Every relationship in the map contributes to the summary weight of a walk through a multiplicative function; this approach is appropriate when the number of factors of the model is pre-set.[45]

In our primary analysis, the between-group comparison will yield three *p* values, and the within group-comparison will yield nine *p* values. We will use the Holm procedure to adjust for multiple testing.[46] The Holm method is as robust as the Bonferroni method, but much more powerful.[47]

Non-statistical methods: the students will enter their narratives of change online. Using AtlasTi 8, two research assistants will individually analyze the transcripts following a deductive thematic analysis approach. In deductive analysis, a theory aligned with the researchers’ interest drives the data analysis;[35] we will use the steps described by the CASCADA model to identify themes of change in the stories.

Missing data: There is no reason a priori to expect differential missing data between game jam and standard lesson groups, since both include a cultural safety educational intervention. We will document missingness and analyze missing data using Amelia II[48] to impute values for missing data with an EM algorithm for the primary outcome. Estimates will reconcile data from ten imputed data sets using Rubin’s approach[49] in the R package Zelig.[50] Additionally, we will provide an attrition diagram[51] (e.g., proportion of participants completing the surveys in each group plotted over time) demonstrating the engagement of participants over time.

### Ethics

This RCT applies the ethical principles in the Tri-Council Policy Statement[52] and was approved by Institutional Review Board of the McGill’s Faculty of Medicine (approval number A05-B37-17B) and by the Sub-committee for Research of the Faculty of Medicine at *La Sabana* University (approval number 445). We will explain the confidentiality and anonymity mechanisms and the voluntary nature of participation, and obtain informed consent of participants prior to the study.

The facilitators will ensure that each participant has signed the online informed consent before proceeding with any research activity. They will be available to explain the purpose of the study, potential risks and benefits, the confidentiality of responses, and the respondents’ rights to not answer certain questions or to end their participation in the study.

## Results

Study enrollment began in July 2019. 640 students completed the baseline survey and were randomised. Data collection is expected to be complete by July 2020 and results are expected in October 2020.

## Discussion

This will be the first medical education RCT using a game jam as an educational intervention. The focus of game jams up to now has been on their products, which are generally video games. Our proposal is to explore the transformative engagement occurring as a result of participating in a game jam.

Answering our research question will advance the current knowledge on game jam research and participatory design in game learning. More important, implementing this project will contribute to the exploration of new strategies to solve the challenges of cultural safety training in medical education, taking into consideration the time pressure in medical studies and the expectations and needs of Millennial medical students.

Some have recently advocated for the need to promote cultural safety rather than cultural competence.[53] To the best of our knowledge, this will be the first initiative using the cultural safety approach in South America. Similarly, cultural safety has been traditionally restricted to the Indigenous context,[54] and this will be one of the first experiences applying cultural safety in a non-Indigenous setting.

Benefits from this project include medical students gaining broader tools for their future work, including openness and dialogue about cultural and traditional health practices. This aspect will be especially relevant for them since most Colombian medical students must work at least one year in a rural area as part of their compulsory one-year return service.

Long-term potential benefits derived from the project include enhanced quality in Colombian health services, improved reputation of health institutions (higher patient satisfaction, better doctor-patient relationship, and better patient adherence), and reduced health disparities among culturally diverse patients in Colombia. Assessing these outcomes is, however, outside the scope of our study.

We recognise several challenges. Participatory design of serious games is an emerging field, and evidence of its impact is scarce.[55] There are no agreed methodological frameworks, nor consensus on operational definitions. This could lead to unexpected challenges, hindering the research process. To address this issue, we conducted a pilot RCT with 79 final-year medical students to explore the acceptability and feasibility of cultural safety training through co-designed game learning, master the skills required to conduct a full-scale co-designed game learning session, pilot research methods and procedures, explore the validity and reliability of our research instrument, and logistical problems that might hinder the full-scale study. This helped us to understand and to solve, in advance, some of the challenges. We will publish the results of the pilot RCT soon.

It is likely that only students interested in cultural safety, game learning, or research will agree to participate in the study. We will implement measures suggested by Kahan[56] to prevent self-selection bias in our study. We will use computerized randomization and all students will have equal probability to be randomised to the intervention or control arm. Although blinding is nearly impossible in RCTs applied to education research, the students will not be aware of the allocation sequence nor what group they were allocated in. All they will know is the auditorium that each of them should attend on the day of the intervention. Our facilitators will prevent students from deliberately switching their allocation status. Finally, five facilitators in each study arm site will ensure that participants remain in their designated groups (game jam or standard lecture).

Some argue that the reproducibility of educational interventions is hard to ensure due to the “specific teacher effect” where the results of an intervention stem from the skills of a particular teacher.[57] To maximize the reproducibility and generalisability of our intervention, we will follow the recommendations provided by the British Medical Journal.[58] This involves describing the intervention rigorously enough to allow its reproducibility and scrutiny in the future. We will report details about the teachers (background, years of experience, fields of expertise) and the teaching interventions (duration, education content, and pedagogical approach).

In this project, we will assess education-related outcomes based on a theory of planned behavior. Experts in cultural safety training recommend, however, the use of patient-related outcomes such as evaluations of care, health outcomes, involvement in care, and health behaviors, to assess cultural safety interventions.[59] Assessing patient-related outcomes would require a more complex approach that goes beyond our logistical and economic capacity. The impact assessment, however, will include a qualitative understanding through the Most Significant Change evaluation. This will document narratives of change in the clinical practice of medical students.

The findings of this project will be specific to the Colombian cultural context. In Colombia, exploring ethnocentrism and cultural safety is simplified by the very widespread use of traditional health practices.[28] In other settings, where cultural and traditional health practices are not widespread, this approach will be less relevant, and it might be necessary to confront ethnocentrism in a more abstract way or through other stigmatizations.

## Acknowledgements

This study was financed by two travel awards awarded to the first author by McGill University: the Norman Bethune Award for Global Health and the Graduate Mobility Award. The first author is supported by the CEIBA Foundation (Colombia) and the Fonds de recherche du Québec – Santé (Canada). This did not influence the design, execution, or publication of the study. Cass Laurie helped proofread the final version of the manuscript and supported its write-up.

## Authors' contributions

This study is part of the PhD work of JP. NA is the supervisor and AC is the co-supervisor of JP. NA is the principal investigator and JP is the study coordinator. NA conceived and advised on the development of the study. AC provided feedback to drafts of the paper. JP drafted this paper and all authors adjusted it. All authors read and approved the final manuscript.

## Conflicts of Interest

None declared.

## Ethics approval and consent to participate

This study was approved by the Institutional Review Board of the McGill’s Faculty of Medicine (approval number A05-B37-17B) and by the Sub-committee for Research of the Faculty of Medicine at *La Sabana* University (approval number 445). Written informed consent was obtained from all participants.

## Abbreviations

RCT: Randomised Controlled Trial

SPIRIT: Standard Protocol Items: Recommendations for Interventional Trials

PICOT: Patients, Intervention, Control, Outcomes, Time-points

TSET-MHP: Transcultural Self- Efficacy Tool—Multidisciplinary Healthcare Provider

CASCADA: **C**onscious knowledge, **A**ttitudes, **S**ubjective norms, **C**hange intention, sense of **A**gency, socialization/**D**iscussion, and behavior change/**A**ction

## References

1. National Aboriginal Health Organization (NAHO). Cultural Competency and Safety: A Guide for Health Care Administrators, Providers and Educators. Health Care (Don Mills). Ottawa, ON; 2008.

2. Kurtz DLM, Janke R, Vinek J, Wells T, Hutchinson P, Froste A. Health Sciences cultural safety education in Australia, Canada, New Zealand, and the United States: a literature review. Int J Med Educ 2018 Oct 25;9:271–285. PMID:30368488

3. Ramsden IM. Cultural Safety and Nursing Education in Aotearoa and Te Wai Pounamu. Nursing (Lond). Victoria University; 2002.

4. Kirmayer LJ. Rethinking cultural competence. Transcult Psychiatry 2012 Apr 16;49(2):149–164. PMID:22508634

5. Maar MA, Beaudin V, Yeates K, Boesch L, Liu P, Madjedi K, Perkins N, Hua-Stewart D, Beaudin F, Wabano MJ, Tobe SW. Wise Practices for Cultural Safety in Electronic Health Research and Clinical Trials With Indigenous People: Secondary Analysis of a Randomized Clinical Trial. J Med Internet Res 2019 Nov 4;21(11):e14203. [doi: 10.2196/14203]

6. Vogel L. Residency programs grapple with new Indigenous cultural safety training requirement. Can Med Assoc J 2018 Jun 25;190(25):E778–E779. PMID:29941440

7. Owens B. Tailoring cultural safety training in health care to local context of Indigenous communities. Can Med Assoc J 2019 Jul 29;191(30):E845–E846. [doi: 10.1503/cmaj.1095780]

8. Guerra O, Kurtz D. Building Collaboration: A Scoping Review of Cultural Competency and Safety Education and Training for Healthcare Students and Professionals in Canada. Teach Learn Med 2017 Apr 3;29(2):129–142. PMID:27813660

9. Thackrah RD, Thompson SC. Applying a Midwifery Lens to Indigenous Health Care Delivery: The Contribution of Campus Learning and Rural Placements to Effecting Systemic Change. Can J Nurs Res 2018 Dec 4;50(4):179–188. [doi: 10.1177/0844562118771829]

10. Dykes DC, White AA. Culturally Competent Care Pedagogy: What Works? Clin Orthop Relat Res 2011 Jul 1;469(7):1813–1816. PMID:21461607

11. Garikapati VM, Pendyala RM, Morris EA, Mokhtarian PL, McDonald N. Activity patterns, time use, and travel of millennials: a generation in transition? Transp Rev 2016 Sep 2;36(5):558–584. [doi: 10.1080/01441647.2016.1197337]

12. Eckleberry-Hunt J, Tucciarone J. The Challenges and Opportunities of Teaching “Generation Y.” J Grad Med Educ 2011 Dec;3(4):458–461. PMID:23205190

13. Mezirow J. Transformative Learning: Theory to Practice. New Dir Adult Contin Educ 1997;1997(74):5–12. PMID:9712154860

14. Taylor EW. An update of transformative learning theory: a critical review of the empirical research (1999–2005). Int J Lifelong Educ 2007 Mar;26(2):173–191. PMID:70199435

15. Fowler A, Ni X (Sherry), Preston J. The Pedagogical Potential of Game Jams. Proc 19th Annu SIG Conf Inf Technol Educ - SIGITE ’18 New York, New York, USA: ACM Press; 2018. p. 112–116. [doi: 10.1145/3241815.3241862]

16. Preston J a, Chastine J, O’Donnell C, Tseng T, MacIntyre B. Game Jams. Int J Game-Based Learn 2012 Jul;2(3):51–70. [doi: 10.4018/ijgbl.2012070104]

17. Chan A-W, Tetzlaff JM, Gotzsche PC, Altman DG, Mann H, Berlin JA, Dickersin K, Hrobjartsson A, Schulz KF, Parulekar WR, Krleza-Jeric K, Laupacis A, Moher D. SPIRIT 2013 explanation and elaboration: guidance for protocols of clinical trials. BMJ 2013 Jan 9;346(e7586):1–42. PMID:23303884

18. La Sabana University. The University in Figures [Internet]. 2018. Available from: https://www.unisabana.edu.co/nosotros/la-sabana-en-cifras/

19. Macklin C, Martin J, Dikkers S. Planning your game jam: game design as a gateway drug. Mob Media Learn Springer-Verlag Publishing; US; 2012. p. 203–18.

20. Pimentel J, Zuluaga G, Isaza A, Molina A, Cockcroft A, Andersson N. Curriculum Co-design for Cultural Safety Training of Medical Students in Colombia: Protocol for a Qualitative Study. In: Costa AP, Reis LP, Moreira A, editors. Comput Support Qual Res Cham, Switzerland: Springer, Cham; 2019. p. 102–109. [doi: 10.1007/978-3-030-01406-3_9]

21. Torgerson DJ, Torgerson CJ. Designing Randomised Trials in Health, Education and the Social Sciences : An Introduction. Palgrave Macmillan. New York, NY: Palgrave Macmillan; 2008. ISBN:9781281976222

22. Andersson N. Building the community voice into planning: 25 years of methods development in social audit. BMC Health Serv Res 2011 Dec 21;11(Suppl 2):S1. PMID:22376121

23. Noar SM, Zimmerman RS. Health Behavior Theory and cumulative knowledge regarding health behaviors: Are we moving in the right direction? Health Educ Res. 2005. PMID:15632099

24. Andersson N, Beauchamp M, Nava-Aguilera E, Paredes-Solís S, Šajna M. The women made it work: fuzzy transitive closure of the results chain in a dengue prevention trial in Mexico. BMC Public Health 2017 May 30;17(S1):408. [doi: 10.1186/s12889-017-4301-0]

25. Dart J, Davies R. A Dialogical, Story-Based Evaluation Tool: The Most Significant Change Technique. Am J Eval 2003 Jun 30;24(2):137–155. [doi: 10.1177/109821400302400202]

26. Schulz KF, Altman DG, Moher D. CONSORT 2010 Statement: Updated Guidelines for Reporting Parallel Group Randomised Trials. PLoS Med 2010 Mar 24;7(3):e1000251. PMID:20352064

27. Institute for Digital Research & Education - UCLA. Power Analysis for Two-group Independent sample t-test | R Data Analysis Examples [Internet]. 2020 [cited 2020 Mar 3]. Available from: https://stats.idre.ucla.edu/r/dae/power-analysis-for-two-group-independent-sample-t-test/

28. World Health Organization. WHO Traditional Medicine Strategy 2002–2005. Geneva, Switzerland; 2002.

29. Eysenbach G, Powell J, Englesakis M, Rizo C, Stern A. Health related virtual communities and electronic support groups : systematic review of the effects of online peer to peer interactions. BMJ 2004;328(May):1–6.

30. Lin C-J, Lee C-K, Huang M-C. Cultural Competence of Healthcare Providers: A Systematic Review of Assessment Instruments. J Nurs Res 2016 Jun;25(3):1. PMID:28481813

31. Pimentel J, Arias A, Ramírez D, Molina A, Chomat A-M, Cockcroft A, Andersson N. Game-Based Learning Interventions to Foster Cross-Cultural Care Training: A Scoping Review. Games Health J 2020 Feb 6;In press:g4h.2019.0078. [doi: 10.1089/g4h.2019.0078]

32. Gozu A, Beach MC, Price EG, Gary TL, Robinson K, Palacio A, Smarth C, Jenckes M, Feuerstein C, Bass EB, Powe NR, Cooper LA. Self-Administered Instruments to Measure Cultural Competence of Health Professionals: A Systematic Review. Teach Learn Med 2007 May 25;19(2):180–190. [doi: 10.1080/10401330701333654]

33. McEldowney R, Connor MJ. Cultural Safety as an Ethic of Care. J Transcult Nurs 2011 Oct 15;22(4):342–349. PMID:21678725

34. Jeffreys MR, Preceded by: Jeffreys MR. Tools for Assessment and Evaluation. Teach Cult competence Nurs Heal care Inq action, Innov Third Edit. Springer Publishing Company; 2015. p. 89–194.

35. Braun V, Clarke V. Using thematic analysis in psychology. Qual Res Psychol 2006 Jan;3(2):77–101. PMID:223135521

36. Basalan I, Temel AB, Chen J, Fridline M, Grund FJ, Halter M et al. Transcultural Self-Efficacy Tool (TSET). In: Jeffreys M, editor. Teach Cult Competence Nurs Heal Care ProQuest Ebook Central; 2015. p. 91–194.

37. Tavakol M, Dennick R. Making sense of Cronbach’s alpha. Int J Med Educ 2011 Jun 27;2:53–55. PMID:28029643

38. Pallant J. SPSS survival manual. Fourth Edi. London, United Kingdom: McGraw-Hill Education; 2010. ISBN:0335208908

39. Davies R, Dart J. The ‘Most Significant Change’ (MSC) Technique. London, United Kingdom; 2005. PMID:19588623

40. Kreuter F, Presser S, Tourangeau R. Social desirability bias in CATI, IVR, and web surveys: The effects of mode and question sensitivity. Public Opin Q 2008;72(5):847–865. PMID:36919497

41. Kiesler S, Siegel J, McGuire TW. Social psychological aspects of computer-mediated communication. Am Psychol 1984;39(10):1123–1134. PMID:18415530

42. Van Gelder MMHJ, Bretveld RW, Roeleveld N. Web-based questionnaires: The future in epidemiology? Am J Epidemiol 2010;172(11):1292–1298. PMID:20880962

43. CIET International. CIET policy on security, storage and destruction of records [Internet]. 2012 [cited 2019 Oct 27]. Available from: http://www.ciet.org/_documents/Policy-records.pdf

44. Yanagawa T, Fujii Y, Mastuoka J. Generalized Mantel-Haenszel Procedures for 2 × J Tables. Environ Health Perspect 1994 Nov;102(suppl 8):57–60. [doi: 10.1289/ehp.94102s857]

45. Niesink P, Poulin K, Šajna M. Computing transitive closure of bipolar weighted digraphs. Discret Appl Math 2013 Jan;161(1–2):217–243. [doi: 10.1016/j.dam.2012.06.013]

46. Holm S. A simple sequentially rejective multiple test procedure. Scand J Stat 1979;(6):65–70.

47. Levin B. On the Holm, Simes, and Hochberg multiple test procedures. Am J Public Health 1996 May;86(5):628–629. [doi: 10.2105/AJPH.86.5.628]

48. Honaker J, King G, Blackwell M. Amelia II: A Program for Missing Data. J Stat Softw 2011;45(7):1–54. PMID:18291371

49. Rubin DB. Multiple imputation for nonresponse in surveys. New York: John Wiley & Sons; 1987. ISBN:0471655740

50. Imai K, King G, Lau O. Zelig: Everyone’s statistical software [Internet]. Gary King. 2009. p. 3–4. Available from: http://gking.harvard.edu/zelig

51. Eysenbach G. CONSORT-EHEALTH: Improving and Standardizing Evaluation Reports of Web-based and Mobile Health Interventions. J Med Internet Res 2011 Dec 31;13(4):e126. PMID:22209829

52. Canadian Institutes of Health Research, Natural Sciences and Engineering Research Council of Canada, Social Sciences and Humanities Research Council of Canada. Tri-Council Policy Statement: Ethical conduct for research involving humans. Med Res Counc Canada. 2014.

53. Curtis E, Jones R, Tipene-Leach D, Walker C, Loring B, Paine S-J, Reid P. Why cultural safety rather than cultural competency is required to achieve health equity: a literature review and recommended definition. Int J Equity Health 2019 Dec 14;18(1):174. [doi: 10.1186/s12939-019-1082-3]

54. Brascoupe S, Waters C. Cultural Safety: Exploring the Applicability of the Concept of Cultural Safety to Aboriginal Health and Community Wellness. J Aborig Heal 2009;5:6–41. PMID:21678725

55. Khaled R, Vasalou A. Bridging serious games and participatory design. Int J Child-Computer Interact 2014 May;2(2):93–100. [doi: 10.1016/j.ijcci.2014.03.001]

56. Kahan BC, Rehal S, Cro S. Risk of selection bias in randomised trials. Trials 2015 Dec 10;16(1):405. [doi: 10.1186/s13063-015-0920-x]

57. Ross S, Loke YK. Do educational interventions improve prescribing by medical students and junior doctors? A systematic review. Br J Clin Pharmacol 2009 Jun;67(6):662–670. PMID:19594535

58. Education Group. Guidelines for evaluating papers on educational interventions. BMJ 1999 Jul 10;319(7202):90–90. PMID:10231261

59. Horvat L, Horey D, Romios P, Kis-Rigo J. Cultural competence education for health professionals. Horvat L, editor. Cochrane Database Syst Rev Chichester, UK: John Wiley & Sons, Ltd; 2014 May 5;CD009405(5):1–100. PMID:24793445

## Multimedia Appendix

**
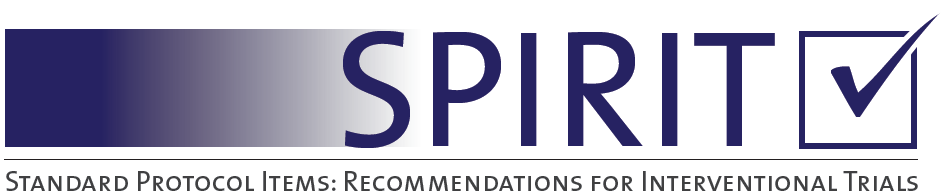
Multimedia Appendix 1. Standard Protocol Items: Recommendations for Interventional Trials (SPIRIT) checklist of the study protocol.**

SPIRIT 2013 Checklist: Recommended items to address in a clinical trial protocol and related documents*

| Section/item | ItemNo | Description | Page Number on which item is reported |
| --- | --- | --- | --- |
| **Administrative information** | | |  |
| Title | 1 | Descriptive title identifying the study design, population, interventions, and, if applicable, trial acronym | 1 |
| Trial registration | 2a | Trial identifier and registry name. If not yet registered, name of intended registry | 3 |
|  | 2b | All items from the World Health Organization Trial Registration Data Set | Appendix |
| Protocol version | 3 | Date and version identifier | Appendix |
| Funding | 4 | Sources and types of financial, material, and other support | 8 and Appendix |
| Roles and responsibilities | 5a | Names, affiliations, and roles of protocol contributors | 8 and Appendix |
|  | 5b | Name and contact information for the trial sponsor | 8 and Appendix |
|  | 5c | Role of study sponsor and funders, if any, in study design; collection, management, analysis, and interpretation of data; writing of the report; and the decision to submit the report for publication, including whether they will have ultimate authority over any of these activities | 8 |
|  | 5d | Composition, roles, and responsibilities of the coordinating centre, steering committee, endpoint adjudication committee, data management team, and other individuals or groups overseeing the trial, if applicable (see Item 21a for data monitoring committee) | N/A |
| Introduction |  |  |  |
| Background and rationale | 6a | Description of research question and justification for undertaking the trial, including summary of relevant studies (published and unpublished) examining benefits and harms for each intervention | 3 to 6 |
|  | 6b | Explanation for choice of comparators | 6 |
| Objectives | 7 | Specific objectives or hypotheses | 6 and 7 |
| Trial design | 8 | Description of trial design including type of trial (eg, parallel group, crossover, factorial, single group), allocation ratio, and framework (eg, superiority, equivalence, noninferiority, exploratory) | 7 and 8 |
| Methods: Participants, interventions, and outcomes | | |  |
| Study setting | 9 | Description of study settings (eg, community clinic, academic hospital) and list of countries where data will be collected. Reference to where list of study sites can be obtained | 8 |
| Eligibility criteria | 10 | Inclusion and exclusion criteria for participants. If applicable, eligibility criteria for study centres and individuals who will perform the interventions (eg, surgeons, psychotherapists) | 8 |
| Interventions | 11a | Interventions for each group with sufficient detail to allow replication, including how and when they will be administered | 9 to 13 |
|  | 11b | Criteria for discontinuing or modifying allocated interventions for a given trial participant (eg, drug dose change in response to harms, participant request, or improving/worsening disease) | 13 |
|  | 11c | Strategies to improve adherence to intervention protocols, and any procedures for monitoring adherence (eg, drug tablet return, laboratory tests) | 13 |
|  | 11d | Relevant concomitant care and interventions that are permitted or prohibited during the trial | 14 |
| Outcomes | 12 | Primary, secondary, and other outcomes, including the specific measurement variable (eg, systolic blood pressure), analysis metric (eg, change from baseline, final value, time to event), method of aggregation (eg, median, proportion), and time point for each outcome. Explanation of the clinical relevance of chosen efficacy and harm outcomes is strongly recommended | 14 and 15 |
| Participant timeline | 13 | Time schedule of enrolment, interventions (including any run-ins and washouts), assessments, and visits for participants. A schematic diagram is highly recommended (see Figure) | 16 |
| Sample size | 14 | Estimated number of participants needed to achieve study objectives and how it was determined, including clinical and statistical assumptions supporting any sample size calculations | 17 |
| Recruitment | 15 | Strategies for achieving adequate participant enrolment to reach target sample size | 18 |
| **Methods: Assignment of interventions (for controlled trials)** | | |  |
| Allocation: |  |  |  |
| Sequence generation | 16a | Method of generating the allocation sequence (eg, computer-generated random numbers), and list of any factors for stratification. To reduce predictability of a random sequence, details of any planned restriction (eg, blocking) should be provided in a separate document that is unavailable to those who enrol participants or assign interventions | 18 |
| Allocation concealment mechanism | 16b | Mechanism of implementing the allocation sequence (eg, central telephone; sequentially numbered, opaque, sealed envelopes), describing any steps to conceal the sequence until interventions are assigned | 19 |
| Implementation | 16c | Who will generate the allocation sequence, who will enrol participants, and who will assign participants to interventions | 18 |
| Blinding (masking) | 17a | Who will be blinded after assignment to interventions (eg, trial participants, care providers, outcome assessors, data analysts), and how | 19 |
|  | 17b | If blinded, circumstances under which unblinding is permissible, and procedure for revealing a participant’s allocated intervention during the trial | N/A |
| **Methods: Data collection, management, and analysis** | | |  |
| Data collection methods | 18a | Plans for assessment and collection of outcome, baseline, and other trial data, including any related processes to promote data quality (eg, duplicate measurements, training of assessors) and a description of study instruments (eg, questionnaires, laboratory tests) along with their reliability and validity, if known. Reference to where data collection forms can be found, if not in the protocol | 19 to 21 |
|  | 18b | Plans to promote participant retention and complete follow-up, including list of any outcome data to be collected for participants who discontinue or deviate from intervention protocols | 22 |
| Data management | 19 | Plans for data entry, coding, security, and storage, including any related processes to promote data quality (eg, double data entry; range checks for data values). Reference to where details of data management procedures can be found, if not in the protocol | 22 and 23 |
| Statistical methods | 20a | Statistical methods for analysing primary and secondary outcomes. Reference to where other details of the statistical analysis plan can be found, if not in the protocol | 23 |
|  | 20b | Methods for any additional analyses (eg, subgroup and adjusted analyses) | 23 and 24 |
|  | 20c | Definition of analysis population relating to protocol non-adherence (eg, as randomised analysis), and any statistical methods to handle missing data (eg, multiple imputation) | 24 |
| **Methods: Monitoring** | | |  |
| Data monitoring | 21a | Composition of data monitoring committee (DMC); summary of its role and reporting structure; statement of whether it is independent from the sponsor and competing interests; and reference to where further details about its charter can be found, if not in the protocol. Alternatively, an explanation of why a DMC is not needed | Appendix |
|  | 21b | Description of any interim analyses and stopping guidelines, including who will have access to these interim results and make the final decision to terminate the trial | Appendix |
| Harms | 22 | Plans for collecting, assessing, reporting, and managing solicited and spontaneously reported adverse events and other unintended effects of trial interventions or trial conduct | Appendix |
| Auditing | 23 | Frequency and procedures for auditing trial conduct, if any, and whether the process will be independent from investigators and the sponsor | Appendix |
| Ethics and dissemination | | |  |
| Research ethics approval | 24 | Plans for seeking research ethics committee/institutional review board (REC/IRB) approval | 24 and 25 |
| Protocol amendments | 25 | Plans for communicating important protocol modifications (eg, changes to eligibility criteria, outcomes, analyses) to relevant parties (eg, investigators, REC/IRBs, trial participants, trial registries, journals, regulators) | Appendix |
| Consent or assent | 26a | Who will obtain informed consent or assent from potential trial participants or authorised surrogates, and how (see Item 32) | 25 |
|  | 26b | Additional consent provisions for collection and use of participant data and biological specimens in ancillary studies, if applicable | N/A |
| Confidentiality | 27 | How personal information about potential and enrolled participants will be collected, shared, and maintained in order to protect confidentiality before, during, and after the trial | 22 and 23 |
| Declaration of interests | 28 | Financial and other competing interests for principal investigators for the overall trial and each study site | 28 |
| Access to data | 29 | Statement of who will have access to the final trial dataset, and disclosure of contractual agreements that limit such access for investigators | 28 |
| Ancillary and post-trial care | 30 | Provisions, if any, for ancillary and post-trial care, and for compensation to those who suffer harm from trial participation | N/A |
| Dissemination policy | 31a | Plans for investigators and sponsor to communicate trial results to participants, healthcare professionals, the public, and other relevant groups (eg, via publication, reporting in results databases, or other data sharing arrangements), including any publication restrictions | Appendix |
|  | 31b | Authorship eligibility guidelines and any intended use of professional writers | Appendix |
|  | 31c | Plans, if any, for granting public access to the full protocol, participant-level dataset, and statistical code | 28 |
| Appendices |  |  |  |
| Informed consent materials | 32 | Model consent form and other related documentation given to participants and authorised surrogates | Available upon request |
| Biological specimens | 33 | Plans for collection, laboratory evaluation, and storage of biological specimens for genetic or molecular analysis in the current trial and for future use in ancillary studies, if applicable | N/A |

*It is strongly recommended that this checklist be read in conjunction with the SPIRIT 2013 Explanation & Elaboration for important clarification on the items. Amendments to the protocol should be tracked and dated. The SPIRIT checklist is copyrighted by the SPIRIT Group under the Creative Commons “[Attribution-NonCommercial-NoDerivs 3.0 Unported](http://www.creativecommons.org/licenses/by-nc-nd/3.0/)” license.

**Appendix**

**2b. World Health Organization Trial Registration Data Set information**

1. Primary Registry and Trial Identifying Number: ISRCTN14261595 (<http://www.isrctn.com>)

[https://doi.org/10.1186/ISRCTN14261595](https://doi.org/10.1186/ISRCTN14261595 )

2. Date of Registration in Primary Registry: July 18^th^, 2019

3. Secondary Identifying Numbers: N/A

4. Source(s) of Monetary or Material Support: McGill University

5. Primary Sponsor: McGill University

6. Secondary Sponsor(s): CEIBA Foundation (Colombia) and Fonds de recherche du Québec – Santé (Canada)

7. Contact for Public Queries: Juan Pimentel (see page 1)

8. Contact for Scientific Queries: Juan Pimentel (see page 1)

9. Public Title: “Game-based learning for improving communication between doctors and communities who use traditional health practices in Colombia”

10. Scientific Title: “Impact of co-designed game learning on cultural safety in Colombian medical education: a randomized controlled trial”

11. Countries of Recruitment: Colombia

12. Health Condition(s) or Problem(s) Studied: see Background section

13. Intervention(s): see Interventions section of Methods

14. Key Inclusion and Exclusion Criteria: see eligibility criteria section of Methods

15. Study Type: Parallel group, two-arm, superiority RCT with 1:1 allocation ratio

16. Date of First Enrollment: July 15^th^, 2019

17. Target Sample Size: 330

18. Recruitment Status: No longer recruiting

19. Primary Outcome(s): see Outcomes section of Methods

20. Key Secondary Outcomes: see Outcomes section of Methods

**Other items from SPIRIT Checklist**

3. Protocol version: 3; last modified July 10th, 2019.

21a, 21b, 22. A full Data Safety Monitoring Board is not necessary for our study. We do not expect our methods to cause any safety concerns to the participants of the trial. However, if there is some distress related to the intervention or control activities, or any other aspect of our methods, the study coordinator will be available to support the participants. We will not conduct interim analyses.

23, 25. The Institutional Review Board of the McGill’s Faculty of Medicine conducts annual reviews of the study procedures and progress. We will communicate any important protocol modification to the Review Board through the Continuing Review Form, which is a mandatory document that we submit each year. This mechanism allows monitoring of changes of financial support, status of the study (enrolment, data analysis, inactive), new risks or benefits for the participants, adverse events, and modifications of the consent form.

31a. We will invite stakeholders (facilitators or medical students) to be co-authors of at least one peer-reviewed manuscript in an open-access journal, such as Games for Health Journal or BMC Medical Education. Similarly, we will invite stakeholders to submit their work and to attend at least two relevant national or international conferences on topics related to the study. Relevant conferences include The North American Primary Care Research Group (NAPCRG) Annual Meeting, The Annual Consortium of Universities for Global Health (CUGH) Global Health Conference, The Unite for Sight Global Health and Innovation Conference, The Cross-Cultural Health Care Conference, and The Society for Intercultural Education, Training and Research (SIETAR) Annual Congress. We will invite end-users (medical students) to develop and circulate a lay report to share findings, reaching all stakeholders.

31b. To define the authorship of the resulting articles of our study, we will follow the Role of Authors and Contributors criteria specified in the Recommendations for the Conduct, Reporting, Editing and Publication of Scholarly Work in Medical Journals of the International Committee of Medical Journal Editors.

**Multimedia Appendix 2. Checklist for Reporting Results of Internet E-Surveys (CHERRIES)**

**Multimedia Appendix 2. Data reporting guidelines, checklist for reporting results of internet E-Surveys (CHERRIES)**

| ***Item category*** | ***Checklist item*** | ***This study*** |
| --- | --- | --- |
| **Design** | Describe survey design | Target population: Undergraduate medical students and medical interns at *La Sabana* University in Colombia.  Purposive sample. We will contact the medical students and medical interns using *La Sabana* University’s mailing lists and will e-mail invitations for voluntary participation in the project. For those willing to participate, we will send further information about the project as well as the date and place of the intervention. We will ask interested students to complete the online informed consent and baseline questionnaire one week before the RCT. |
| **IRB approval and informed consent process** | IRB approval | This RCT was approved by Institutional Review Board of the McGill’s Faculty of Medicine (approval number A05-B37-17B) and by the Sub-committee for Research of the Faculty of Medicine at La Sabana University (approval number 445). |
|  | Informed consent | Informed consent was provided by completing the e-survey. |
|  | Data protection | SurveyMonkey and Google responses are stored in a worksheet that can only be accessed through an account login. Data transmission uses Secure Sockets Layer to encrypt information during transport. The data storage is as secure as most other systems that store survey information. After downloading the data, we will delete it from SurveyMonkey and Google Forms. We will be responsible for ensuring the data are securely stored for seven years and then destroyed in accordance with CIET guidelines for security, storage, and eventual destruction of data records. |
| **Development and pre-testing** | Development and testing | We will use a 30-item instrument comprised of three parts. The first part (five items) will explore sociodemographic factors of the students. The second part (15 items) will be based on the Transcultural Self–Efficacy Tool and will explore knowledge, attitudes, skills, and behaviors of cultural competence. For the third part of the instrument (cultural safety), we developed a Likert-type preliminary version based on our CASCADA variables (see primary outcome) and piloted it for validity and reliability in our pilot RCT.  Regarding the qualitative data, using a pre-defined format in Google forms, we will ask participants to write down and enter their stories. |
| **Recruitment process and description of the sample having access to the questionnaire** | Open survey versus closed survey | Closed survey sent by institutional email from *La Sabana* University. |
|  | Contact mode | Contact with participants online through e-mail and face-to-face. |
|  | Advertising the survey | Not applicable. |
| **Survey administration** | Web/E-mail | E-mail survey using SurveyMonkey and Google Forms. There was an automatic method for capturing responses. |
|  | Context | We will contact the medical students and medical interns using *La Sabana* University’s mailing lists and will e-mail invitations for voluntary participation in the project. |
|  | Mandatory/voluntary | Voluntary survey. |
|  | Incentives | Participants of the qualitative component of the study will enter a raffle after submitting their story. We will offer one gift card of CAD 20 for every ten stories collected. |
|  | Time/date | July 2019 to July 2020. |
|  | Randomization of items or questionnaires | Stratified randomization by cultural safety score at baseline. |
|  | Adaptive questioning | Not applicable. |
|  | Number of items | 30 items in total. |
|  | Number of screens (pages) | Three Webpage per survey. |
|  | Completeness check | We will use several validation options to increase the quality of the data: specific number range, specific character range, date validation, email address format, and prompts that alert participants when they enter incomplete or invalid answers. |
|  | Review step | Respondents were not able to review and change their answers. |
| **Response rates** | Unique site visitor | Repeat visitors will not be excluded. Some students will share their devices with students who do not have access to an electronic device. |
|  | View rate (ratio of unique survey visitors/unique site visitors) | Not available. |
|  | Participation rate (ratio of  unique visitors who agreed to participate/unique first  survey page visitors) | Not available. |
|  | Completion rate (ratio of users who finished the survey/users who agreed to participate) | Not available. |
| **Preventing multiple entries from the same individual** | Cookies used | Repeat visitors will not be excluded. |
|  | IP check | Repeat visitors will not be excluded. |
|  | Log file analysis | Repeat visitors will not be excluded. |
|  | Log file analysis | Repeat visitors will not be excluded. |
|  | Registration | Repeat visitors will not be excluded. |
| **Analysis** | Handling of incomplete questionnaires | Only completed questionnaires will be analyzed. |
|  | Questionnaires submitted with an atypical timestamp | Not applicable |
|  | Statistical correction | Not applicable |
