# Supplementary material for: The most significant change for Colombian medical trainees going transformative learning on cultural safety: qualitative results from a randomised controlled trial
Source: BMC Med Educ. 2022 Sep 10;22:670. doi: 10.1186/s12909-022-03711-1 (PMC9463722; doi:10.1186/s12909-022-03711-1)
Supplement: Supplementary file 2 — Additional file 2. Standards for Reporting Qualitative Research checklist. [file 12909_2022_3711_MOESM2_ESM.docx]

**Additional file 2. Standards for Reporting Qualitative Research checklist^[[1]](#footnote-1)^**

| **No.** | **Topic** | **Item** | **Page** |
| --- | --- | --- | --- |
| **Title and abstract** | | | |
| **S1** | Title | Concise description of the nature and topic of the study Identifying  the study as qualitative or indicating the approach (e.g., ethnography,  grounded theory) or data collection methods (e.g., interview, focus  group) is recommended | 1 |
| **S2** | Abstract | Summary of key elements of the study using the abstract format of  the intended publication; typically includes background, purpose,  methods, results, and conclusions | 2 |
| **Introduction** | | | |
| **S3** | Problem formulation | Description and significance of the problem/phenomenon studied;  review of relevant theory and empirical work; problem statement | 3 & 4 |
| **S4** | Purpose or research question | Purpose of the study and specific objectives or questions | 4 |
| **Methods** | | | |
| **S5** | Qualitative approach and research paradigm | Qualitative approach (e.g., ethnography, grounded theory, case study,  phenomenology, narrative research) and guiding theory if appropriate;  identifying the research paradigm (e.g., postpositivist, constructivist/  interpretivist) is also recommended; rationale^b^ | Study design - 4 |
| **S6** | Researcher characteristics and reflexivity | Researchers’ characteristics that may influence the research, including  personal attributes, qualifications/experience, relationship with  participants, assumptions, and/or presuppositions; potential or actual  interaction between researchers’ characteristics and the research  questions, approach, methods, results, and/or transferability | Rigour - 6 |
| **S7** | Context | Setting/site and salient contextual factors; rationale^b^ | Setting and participants – 4 & 5 |
| **S8** | Sampling strategy | How and why research participants, documents, or events were  selected; criteria for deciding when no further sampling was necessary  (e.g., sampling saturation); rationale^b^ | Setting and participants – 4 & 5, and Data collection – 5 & 6 |
| **S9** | Ethical issues pertaining to human subjects | Documentation of approval by an appropriate ethics review board  and participant consent, or explanation for lack thereof; other  confidentiality and data security issues | Ethical approval - 7 |
| **S10** | Data collection methods | Types of data collected; details of data collection procedures including  (as appropriate) start and stop dates of data collection and analysis,  iterative process, triangulation of sources/methods, and modification  of procedures in response to evolving study findings; rationale^b^ | Data collection – 5 & 6 |
| **S11** | Data collection instruments and technologies | Description of instruments (e.g., interview guides, questionnaires)  and devices (e.g., audio recorders) used for data collection; if/how the  instrument(s) changed over the course of the study | Data collection – 5 & 6 |
| **S12** | Units of study | Number and relevant characteristics of participants, documents, or  events included in the study; level of participation (could be reported  in results) | Data processing and analysis - 6 |
| **S13** | Data processing | Methods for processing data prior to and during analysis, including  transcription, data entry, data management and security, verification  of data integrity, data coding, and anonymization/deidentification of  excerpts | Data processing and analysis - 6 |
| **S14** | Data analysis | Process by which inferences, themes, etc., were identified and  developed, including the researchers involved in data analysis; usually  references a specific paradigm or approach; rationale ^b^ | Data processing and analysis - 6 |
| **S15** | Techniques to enhance trustworthiness | Techniques to enhance trustworthiness and credibility of data analysis  (e.g., member checking, audit trail, triangulation); rationale^b^ | Rigour - 6 |
| **Results/findings** | | | |
| **S16** | Synthesis and interpretation | Main findings (e.g., interpretations, inferences, and themes); might  include development of a theory or model, or integration with prior  research or theory | Results - 7 to 12 |
| **S17** | Links to empirical data | Evidence (e.g., quotes, field notes, text excerpts, photographs) to  substantiate analytic findings | Additional file 2. Deductive thematic analysis results - complete list of quotes |
| **Discussion** | | | |
| **S18** | Integration with prior work, implications,  transferability, and contribution(s) to the field | Short summary of main findings; explanation of how findings  and conclusions connect to, support, elaborate on, or challenge  conclusions of earlier scholarship; discussion of scope of application/  generalizability; identification of unique contribution(s) to scholarship  in a discipline or field | Discussion 12 and 13 |
| **S19** | Limitations | Trustworthiness and limitations of findings | Limitations - 13 |
| **Other** | | | |
| **S20** | Conflicts of interest | Potential sources of influence or perceived influence on study conduct  and conclusions; how these were managed | 14 & 15 |
| **S21** | Funding | Sources of funding and other support; role of funders in data  collection, interpretation, and reporting | 14 & 15 |

^a^ The authors created the SRQR by searching the literature to identify guidelines, reporting standards, and critical appraisal criteria for qualitative research; reviewing the reference lists of retrieved sources; and contacting experts to gain feedback. The SRQR aims to improve the transparency of all aspects of qualitative research by providing clear standards for reporting qualitative research.

^b^ The rationale should briefly discuss the justification for choosing that theory, approach, method, or technique rather than other options available, the assumptions and limitations implicit in those choices, and how those choices influence study conclusions and transferability. As appropriate, the rationale for several items might be discussed together.

1. O’Brien, B. C., Harris, I. B., Beckman, T. J., Reed, D. A., & Cook, D. A. (2014). Standards for Reporting Qualitative Research. Academic Medicine, 89(9), 1245–1251. https://doi.org/10.1097/ACM.0000000000000388 [↑](#footnote-ref-1)
