# Supplementary material for: The most significant change for Colombian medical trainees going transformative learning on cultural safety: qualitative results from a randomised controlled trial
Source: BMC Med Educ. 2022 Sep 10;22:670. doi: 10.1186/s12909-022-03711-1 (PMC9463722; doi:10.1186/s12909-022-03711-1)
Supplement: Supplementary file 3 — Additional file 3. Deductive thematic analysis results - complete list of quotes. [file 12909_2022_3711_MOESM3_ESM.docx]

**Additional file 3. Deductive thematic analysis results - complete list of quotes**

| **1. CONSCIOUS KNOWLEDGE** |
| --- |
| **1.1. Benefits of cultural safety training and consequences of cultural risk**  *Benefits – adherence* "[cultural safety] helps to reach consensus between patients and me, it makes patients feel involved in their treatment without feeling diminished, therefore strengthening their adherence to medical treatment." (Participant 7) |
| *Benefits – positive environment* “generating a pleasant and happy environment in which the patient feels that I take his culture into account and that, together, we can achieve positive health outcomes.” (Participant 10) |
| *Benefits - doctor-patient relationship* “Taking the time to investigate about the traditional practices of patients allows you to gain important knowledge and to develop a better relationship with patients.” (Participant 16) |
| *Consequences of cultural risk* "Diminishing or discriminating against someone for their beliefs or for using traditional medicine is like attacking that person's identity and culture." (Participant 4) |
| *Issues approaching patients “*We were doing surveys about breastfeeding practices, and one of the questions was literally about ‘breastfeeding myths’. Shortly after starting the survey, we realized that it was a very poorly formulated question, mothers did not understand about ‘myths’, simply because for them they were not myths.*”* (Participant 9) |
| **1.2. Acknowledging cultural diversity and traditional medicine use among family, student setting, and the student themselves** |
| *Traditional medicine use among family and students* "In the lecture, we learned about remote tribes and communities, but I don't have to go that far to experience what I learned from the talk. Not only my dad uses traditional medicine, but most of my family, even me. For example, I drink cinnamon infusion when I have a colic. ” (Participant 19) |
| *Traditional medicine use in my setting* “Being able to accept that there is a diversity of knowledge […] is a great contribution to our clinical practice throughout the semester. We have the opportunity to work in Cundinamarca [Colombian province] where the rural population tends to have a rich oral tradition to manage their health” (Participant 26) |
| *Cultural diversity* "The most significant change I had was facing a ‘new country’ and realizing that there are many ways to practice medicine that are not taken into account in our medical education" (Participant 2)  "I identify more different cultures and I appreciate much more the importance that this has on my medical practice" (Participant 11) |
| *Traditional medicine is part of our culture* "The most important thing is to recognize and respect cultural differences and protect them. Above all, preserve cultural practices and transmit them on from generation to generation, as this is part of the history of our region." (Participant 25) |
| **1.3. Characteristics of traditional medicine and confusion of concepts** |
| “I can now differentiate the concept of alternative medicine as from traditional medicine. I understood that traditional medicine has a way of being and that it is part of someone else's culture. ” (Participant 4) |
| *Confusion with homeopathy* "Because the temptation when seeing a patient is to deny that homeopathic medicine will have any beneficial effect on our patients." (Participant 18) |
| **2. ATTITUDES** |
| **2.1. Respect and appreciation for cultural diversity, and avoid cultural destruction and ethnocentrism** |
| *Acceptance of traditional medicine* "Cultural safety training has allowed me to accept the traditional ways that do not correspond to western medicine" (Participant 16) |
| *Respect and cultural preservation* "The most important thing is to recognize and respect cultural differences and protect them. Above all, preserve cultural practices and transmit them on from generation to generation, as this is part of the history of our region." (Participant 25) |
| *Avoid discrimination* "[Cultural safety] taught me that we should not belittle traditional medicine or believe that Western medicine is the truth and the solution to everything." (Participant 23) |
| *Appreciate cultural diversity* "Now I can identify different cultures and I appreciate much more the importance that cultural diversity has on my medical practice" (Participant 24) |
| *Less ethnocentrism* "Because when we find a patient who has a culture with different beliefs from ours, we tend to ignore them; we believe that our culture is correct and we that are right, but [cultural safety training] make us wonder if it really like that." (Participant 21) |
| **2.2. Openness** |
| “As doctors in training, we often think that only our knowledge is objective and reasonable, but we must open our minds to accept the cultural practices and beliefs of other people and other communities” (Participant 25)  " I am now much more open to recognize and accept the cultural differences that my patients may have" (Participant 13)  "[Cultural safety] training allows me to have an open mind to the beliefs and cultural practices that patients have; [It allows me to] take these aspects into account to prevent judgments and even to learn from them, from their experience" (Participant 7) |
| **2.3. Self-awareness, cultural awareness, and awareness of benefits** |
| *Cultural awareness* "Now I understand that all of us, including the patients, grow with different customs, needs, and beliefs, and that all of us belong to a culture and therefore to a different way of seeing diseases and their treatment" (Participant 21)  "[Cultural safety] allowed me to understand that traditional medicine and culture is a lifestyle that has endured in the history of humanity for centuries... Long before the arrival of Western medicine. As a medical student I am biased and on various occasions I have confronted the culture of my patients." (Participant 23) |
| *Self-awareness* "I am aware of the paradigms and prior knowledge that I have from my training that can influence my clinical practice; it allowed me to be more empathetic and assertive when interacting with my patients" (Participant 1)  "Participating in this activity gives us a broader perspective; it allows us to realize that often we want to impose our will on the patient" (Participant 3) |
| **3. SUBJECTIVE NORMS** |
| **3.1. Positive perception of traditional medicine, respect for patients, and eluding cultural risk** |
| *I respected traditional practices* “Now I see the impact that cultural practices have on patients' perception of health. I consider what people say about their beliefs, before I didn't even pay attention to it” (Participant 9) |
| *Avoid cultural risk* “Often we want to impose our will on the patient; they might refuse to follow a treatment because they consider that physicians despise their ideas and culture. On the other hand, understanding and trying to include the patients' culture in the treatment of any pathology could lead to better health outcomes for the patient.” (Participant 21) |
| *Classic training* "It showed me that one should not reject cultural practices just because they are not part of classic medical education" (Participant 16) |
| **3.2. Biomedical model and evidence-based medicine** |
| "[Cultural safety] taught me that we should not belittle traditional medicine or believe that Western medicine is the truth and the solution to everything." (Participant 23)  “In retrospect, the patient felt that traditional medicine worked for him, even more than the medication he was taking, and I had no right to tell him that traditional practices had no evidence and therefore, he had to stop doing them.” (Participant 8) |
| **3.3. Acknowledge benefits of cultural safety training and less ethnocentrism** |
| *Ethnocentrism* "Because when we find a patient who has a culture with different beliefs from ours, we tend to ignore them; we believe that our culture is correct and we that are right, but [cultural safety training] make us wonder if it really like that." (Participant 21)  “As doctors in training, we often think that only our knowledge is objective and reasonable, but we must open our minds to accept the cultural practices and beliefs of other people and other communities” (Participant 25) |
| *Other physicians don´t have time* "I think that at another time, I could say that [traditional practices] are absurd. Many doctors only think about imposing their opinion either because they are not interested or because the office time is short; they don't take the time to listen to their patients and much less to discuss with the patient in order to improve their health. ” (Participant 6) |
| *Overcoming challenges* "A situation that previously could have represented an obstacle and a reason for not carrying out treatment is now an opportunity to make the patient feel safe and to prevent judging or discriminating against them." (Participant 13)  “Before it was an unpleasant experience. There were many occasions in which the grandmothers talked about home remedies whose names were difficult for me to understand (I am not from this region); I doubted their effectiveness. Today, those experiences are not unpleasant anymore. I can now learn from the context of my patient, therefore providing better healthcare.” (Participant 17) |
| **4. CHANGE INTENTION** |
| "We were overwhelmed by the situation: we had a minor patient who was a member of a community that was different from ours, accompanied by his father who did not allow us to touch his son, did not speak or understand our language. For a moment all that seemed like a barrier, but I remembered what I had learned and decided to try to make the change that I would like to see if I were that patient” (Participant 13)  " I feel that this is what I want to do in my professional practice, to be able to help my patients, understand that traditional medicine is not wrong, and that [cultural safety] can enhance the doctor-patient relationship." (Participant 5) |
| **5. Agency** |
| **5.1. Able to accept cultural diversity in health care** |
| "I am able to accept that there are diversity of beliefs and that each one has its cultural and scientific basis. It is a great contribution to our clinical practice." (Participant 26) |
| **5.2. Able to prevent culturally unsafe actions and improve the doctor-patient relationship** |
| "I am able to improve the doctor-patient relationship. I can now create a relationship with patients from different cultures without imposing my thoughts, making judgments, or demeaning my patients and their families." (Participant 7)  "Currently, I am open to recognize and accept the cultural differences that my patients may have. I am always trying not to make them feel judged or discriminated against because of their beliefs or their culture" (Participant 25) |
| **6. DISCUSSION** |
| “I listened to the patients more; we discussed ways to take care of children, comparing things that they believed and had done with things that we knew from our medical knowledge; it helped to improve the connection with patients to ensure proper growth and development of babies. This was quite special because the nursing students who rotated with us also supported our approach, and we learned from each other. ” (Participant 1) |
| **7. ACTION** |
| **7.1. Better communication and relationship with patients and with other health professionals** |
| *Better communication, assertiveness, and empathy* "I am aware of the paradigms and prior knowledge that I have from my training that can influence my clinical practice; it allowed me to be more empathetic and assertive when interacting with my patients" (Participant 1) |
| *Doctor-patient relationship and between health professionals* “I listened to the patients more; we discussed ways to take care of children, comparing things that they believed and had done with things that we knew from our medical knowledge; it helped to improve the connection with patients to ensure proper growth and development of babies. This was quite special because the nursing students who rotated with us also supported our approach, and we learned from each other. ” (Participant 1) |
| **7.2. Better outcomes for patients, physicians, and society** |
| *Patients feel more understood, safe, and cared for, and it humanizes medicine* “Things that may seem so simple, but that for someone like the patient in the story, are important things. [Cultural safety] makes people feel safer, understood, and cared for, and they feel the desire to come back to see a doctor who also cares for them; in my opinion, it touches the most human part of medical practice. ” (Participant 15) |
| *Cultural preservation* “The patient was very frustrated by her family situation, but fortunately I was able to calm her down and say that her mother did all this because she loved her and her grandson. I told her that, although all this seemed very strange to her, her mother had years of experience in these subjects [traditional medicine] and that, although we could take care of her using modern medicine, everything that her mother gave her was care that we could not offer. I asked her to please be patient with her mother because she knows her daughter and her culture more than ourselves.” (Participant 20) |
| *More knowledge* "Because it showed me that one should not reject [traditional medicine] just because it is not part of classic medical education. Taking the time to investigate [traditional medicine] allows gaining important knowledge and developing a better relationship with the patient." (Participant 16) |
| **7.3. Dialogue and integration/balance/consensus between traditional medicine and modern medicine** |
| *Balance and integration between traditional medicine and modern medicine* “I listened to the patients more; we discussed ways to take care of children, comparing things that they believed and had done with things that we knew from our medical knowledge.” (Participant 1)  *Dialogue and consensus* "The grandmother understood that there are times where they should go to the hospital while I did not prohibit her practices for handling these situations at home. We were able to reach a consensus in which she understands that it is okay to go to the hospital in certain situations and I did not prevent her from continuing to use her traditional practices. Moreover, I learned about a home-made way of managing acute diarrhea” (Participant 7)  “I told her that, although all this seemed very strange to her, her mother had years of experience in these subjects [traditional medicine] and that, although we could take care of her using modern medicine, everything that her mother gave her was care that we could not offer.” (Participant 17) |
| *Health care decision-making process* “After several minutes explaining what the treatment was, the father accepted and asked us if he could continue giving his son the herbal tea and applying the cream that his wife had prepared with medicinal plants. We asked him what plants they were using. We investigated and discovered that several plants had anti-inflammatory properties. In the end, the agreement was that they had to come to the change of plasters every 8 days, and he could use the remedies. The father felt calmer knowing that he could continue using his own medicines” (Participant 13) |
| *I shared my knowledge and point of view* "I was also able to share what was best for the child from my point of view, and when to go to the emergency services to avoid complications in the future, without attacking, scolding or diminishing the traditional practices of the grandmother" (Participant 7)  “They told us that since they arrived in Colombia it had been very difficult for them to find their medicinal plants. They have different names in Venezuela. Therefore, when they had a health issue they had to agree to the use of drugs, which they do not like. The doctor explained the importance of the drugs; however, we also suggest some plants that could be similar to those that they used in his country. She was very pleased with what the medical team had done.” (Participant 14) |
| **7.4. I explored and investigated about traditional medicine, and listened and learned from patients** |
| *Curiosity about traditional practices* “During my Gynecology and Obstetrics rotation at Kennedy’s Hospital, it was common in prenatal check-ups to hear from several expectant mothers, talking about the use of *brevo* [medicinal plant] leaf baths. That was totally unknown to me at the time. I knew that *brevo* was the tree where the *brevas* grow [fruits that are traditionally eaten with *arequipe*]. I generally told the moms that the *brevo* baths were not necessary, that they were useless. However, while I was learning about cultural safety, I asked a patient the reason for these baths, to which she replied, ‘it is that they serve to be able to start contractions.’ After the consultation, I investigated about the remedy and found that indeed the *brevo* leaf is used to start labor. Of course, I only knew of oxytocin and misoprostol, I had never heard of this practice, which as I read has been used since many, many years ago here in Colombia.” (Participant 20)  "when we asked the patients about traditional remedies, many told us about how drinking *hinojo* [fennel] tea increase milk production." (Participant 9) |
| *I learned about traditional medicine* "I was able to learn something that is not found in the so-called "evidence " upon which medicine is based so much today. I learned about a traditional practice passed down through generations" (Participant 7)  “I am now more willing to listen to the patients regarding the way they understand disease, what they have, and what they believe help them and does them good. In this way, I have also been able to learn from them and their traditions." (Participant 15)  “We were able to reach a consensus in which she understands that it is okay to go to the hospital in certain situations and I did not prevent her from continuing to use her traditional practices. Moreover, I learned about a home-made way of managing acute diarrhea” (Participant 7) |
| *I investigated about the cultural context* “A month ago, while rotating in the children's orthopedic department at the Roosevelt Institute, a 13-year-old patient from an Indigenous community came to us. They are the *Piapocos* and their community is located between the *Meta* and *Guaviare* rivers […] The first thing I did was investigate a little bit about that community, about their beliefs, traditions, how was their organization. I wanted to know how to best approach them.” (Participant 13) |
| *I investigated about traditional medicine* “Six months ago, when I was rotating in the general surgery department at the University of La Sabana Clinic, a patient came with an ulcer that was being treated with honey. At first, I thought it was not the ideal management, however reading about cures I found that honey has many properties that help wound healing” (Participant 16) |
